# Automated Behavioral Experiments in Mice Reveal Periodic Cycles of Task Engagement within Circadian Rhythms

**DOI:** 10.1523/ENEURO.0121-19.2019

**Published:** 2019-09-17

**Authors:** Nikolas A. Francis, Kayla Bohlke, Patrick O. Kanold

**Affiliations:** Department of Biology, University of Maryland, College Park, Maryland 20742

**Keywords:** auditory, circadian, high throughput, home cage, operant conditioning

## Abstract

High-throughput automated experiments accelerate discovery in neuroscience research and reduce bias. To enable high-throughput behavioral experiments, we developed a user-friendly and scalable automated system that can simultaneously train hundreds of mice on behavioral tasks, with time-stamped behavioral information recorded continuously for weeks. We trained 12 cages of C57BL/6J mice (24 mice, 2 mice/cage) to perform auditory behavioral tasks. We found that circadian rhythms modulated overall behavioral activity as expected for nocturnal animals. However, auditory detection and discrimination accuracy remained consistently high in both light and dark cycles. We also found a periodic modulation of behavioral response rates only during the discrimination task, suggesting that the mice periodically reduce task engagement (i.e., take “breaks”) when task difficulty increases due to the more complex stimulus–response paradigm for discrimination versus detection. Our results highlight how automated systems for continuous high-throughput behavioral experiments enable both efficient data collection and new observations on animal behavior.

## Significance Statement

Automated high-throughput behavioral experiments in mice promise researchers the ability to quickly and reliably asses the behavior of large animal populations, while also minimizing experimenter-induced bias. However, the technical complexities of automation have limited widespread adoption of high-throughput behavioral methods. Here, we present a new tool for behavioral research, the ToneBox, which allows both novice and expert behaviorists to automatically test hundreds of mice simultaneously on different behavioral tasks. We provide manufacturing specifications and detailed documentation of system operation. Using C57BL/6J mice, we show that the ToneBox tracks circadian cycles via behavioral response rates, and that task difficultly modulates duty cycles of task engagement.

## Introduction

Targeted investigation of the links between genetics, the brain, and behavior has seen rapid advancement because of powerful new tools for high-throughput experiments (Rose et al., 2016; Sofroniew et al., 2016; Kuchibhotla et al., 2017; Francis et al., 2018). Automated behavioral experiments (Gess et al., 2011; Schaefer and Claridge-Chang, 2012; Poddar et al., 2013; de Hoz and Nelken, 2014; Francis and Kanold, 2017; Balzani et al., 2018) in mice will enable the advancement of neuroscience research by efficiently producing large high-quality datasets that bypass the limitations of manual behavioral methods, such as daily animal handling at suboptimal times during the circadian rhythm—which may stress animals and increase behavioral variability across experiments (Balcombe et al., 2004). Automation removes experimenter-induced variability and makes more efficient use of both human and animal resources. However, technical complexities such as calibrated stimulus presentation, scalable automation, and big data analysis have limited the widespread adoption of high-throughput automated behavioral methods across neuroscience laboratories.

Given the potential for great scientific advancement, we developed the “ToneBox,” a scalable and user-friendly automated system for 24/7 behavioral training in mice within their home cage. The ToneBox does not require new users to have deep technical knowledge to build and operate it, yet the ToneBox also enables accurate stimulus calibration and informative analysis of large behavioral datasets. ToneBox specifications and operation are detailed within both the Materials and Methods and an on-line repository. The repository contains a manual with parts lists and comprehensive step-by-step instructions for assembly and operation. We also provide models for 3D printing and manufacturing of printed circuit boards (PCBs), as well as software for system operation and data analysis.

Using the ToneBox, we found that while circadian rhythms modulated overall behavioral activity, as expected for nocturnal animals such as mice, auditory detection and discrimination accuracy remained consistently high in both light and dark cycles. We compared task performance during an easy task (pure-tone detection) versus a hard task (pure-tone frequency discrimination) and found a periodic modulation of behavioral response rates only during the more difficult task.

## Materials and Methods

### Animals

Twenty-four C57BL/6J mice (12 male, 12 female) were trained simultaneously on auditory tasks in 12 home cages (two mice per cage). Mice were received at postnatal day 30 (P30). All mice were maintained on a 12 h (5:00 P.M. to 5:00 A.M.) light cycle and a 12 h (5:00 A.M. to 5:00 P.M.) dark cycle, and were provided food *ad libitum*. The mice were visually inspected every day (see below), and cages were cleaned weekly. All experimental procedures were approved by the University of Maryland Animal Care and Use Committee.

### Hardware

**Figure 1.**
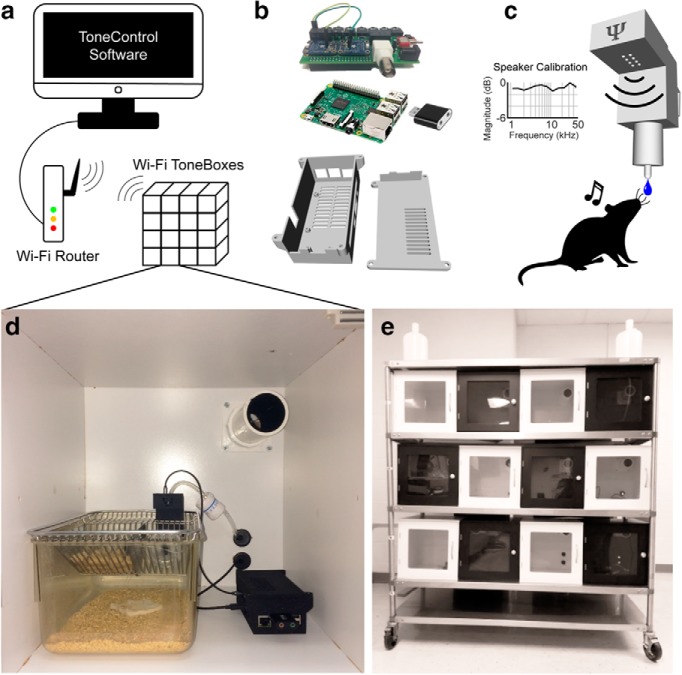
The ToneBox system for automated auditory operant conditioning in the mouse home cage. ***a***, ToneControl software runs from the Matlab environment on a desktop computer. A Wi-Fi router is used to connect the computer to each Wi-Fi ToneBox. ***b***, The ToneBox CCU. Bottom, A 3D model of the CCU case. Inside each CCU is a Raspberry Pi 3+ (middle) that is connected to a custom shield (top) with two capacitive touch sensors used to monitor behavioral responses. A USB sound card (middle) is used for audio input and output. ***c***, The ToneBox BI: 3.5 mm audio cables connect the CCU to the BI within each home cage. An overhead speaker in the BI presents sounds. Pure tones of 1-45 kHz were calibrated to <1 dB magnitude variability. The waterspout at the base of the BI is connected to the CCU capacitive touch sensor to detect licks. ***d***, The CCU, BI, and home cage are placed within an actively ventilated enclosure. The BI is mounted on the side of the cage. The BI is designed to place the waterspout inside the cage, while keeping the speaker outside to avoid damage from exploratory behavior by the mice. Water tubing enters through the back of the enclosure, connecting a flow regulator to the waterspout. ***e***, Twelve enclosures with clear doors are shown stacked together on a mobile rack, with water supplies.

Each ToneBox (Fig. 1) consists of the following seven components: (1) a computer with Matlab and a Wi-Fi router; (2) a ToneBox central control unit (CCU) with Raspberry Pi and custom ToneBox shield; (3) a USB (universal serial bus) sound card; (4) a behavioral interface (BI) that contains a speaker and a touch-responsive waterspout; (5) a sound attenuating box; (6) a home cage; and (7) a water delivery system with solenoid valves. A detailed list of parts, 3D models, a PCB file, and assembly instructions are available in the on-line public repository.

In brief, the CCU connects to both the BI and water delivery system via 3.5 mm TRS (tip, ring, and sleeve) cables. The Raspberry Pi is powered by a micro USB cable, and connected to a computer using a local Wi-Fi network. The speaker in the BI is driven by a 2.5 W class D audio amplifier (catalog #PAM8302, Adafruit) that receives audio from the USB audio device connected to the CCU. Water delivery is controlled by a normally closed 12 V solenoid valve (catalog #BCBI6683, American Science & Surplus) that is connected to a water reservoir through a manual valve. The solenoid connects to the CCU using a TRS cable. Since USB 2.0 only provides 5 V power, a 12 V input to the CCU is connected to a relay switch controlled by the Raspberry Pi. The CCU also contains capacitive touch sensors (catalog #AT42QT1012, Adafruit) that receive input from a TRS cable connected to the waterspout in the BI. The BI consists of two main components: a water-resistant speaker for sound delivery (catalog #AS02708CO-WR-R, PUI Audio), and a custom 3D printed stainless steel waterspout. The BI is hung on the side of a standard 13 × 7 × 5 inch home cage using a clip on the back of the BI. A small rectangle the size of the BI must be cut out of the cage lid to fit the BI in the home cage. The BI speaker was calibrated *in situ* using a Brüel & Kjær 4944-A microphone. Calibration was performed by whitening the recording of a 1-45 kHz noise played through the speaker, then applying the whitening filter to pure tones used in the task (Fig. 1*c*). The BI, CCU, and home cage sit within a sound-attenuating chamber. This is simply a cabinet with a clear door and a ventilating fan. The cabinets provide ≥20 dB attenuation between ToneBoxes for tones >1 kHz, allowing simultaneous auditory training to be possible for multiple cages. Greater attenuation is possible with additional sound-proofing customization of the cabinets. The waterline is run from the source, into the cabinet, and then finally down to the waterspout inside the BI. We placed 12 ToneBoxes on a metal rack inside our animal housing facility, so that experiments could run 24 h/d.

### Software

The ToneBox is a hardware system that can be controlled by any software that has drivers for the Raspberry Pi 3B. Here, we ran the ToneBox on a Windows 10 PC with a custom GUI (graphical user interface; “ToneControl”) written in the Matlab programming language (MathWorks). The user can select up to 16 ToneBoxes, although the software could be customized for more ToneBoxes. After selecting a ToneBox, the user chooses a training phase: habituation, shaping, detection, or discrimination. Next, the user selects the target and nontarget (for discrimination) pure-tone frequencies and levels. Silent probe trials can also be selected. Before starting experiments, the user can test the water and sound presentation using GUI buttons. In addition, the user can choose to calibrate the speaker.

We also developed a “ToneGraph” GUI for data analysis and for monitoring system status. The user can plot all data from each cage separately or take averages across cages. The user can also select subsets of trials for analysis. ToneControl, ToneGraph, and a software manual are available in the on-line public repository together with a test dataset to learn its function.

### Behavioral training

For our initial experiments, we trained 12 cages of mice (two mice per cage) simultaneously. Individual mice, and a cage of 4 mice were also trained. Each group of cage mates was trained using positive reinforcement to detect a pure tone to receive water. Cages were housed inside sound-attenuating chambers that were arranged on shelves in our animal housing facility. Training consisted of the following four sequential phases: habituation, shaping, detection, and discrimination. Water bottles were removed from each cage, and the mice were water restricted for 23 h before habituation. To motivate task acquisition, water was made available only through task performance. Training ran on a 1 h duty cycle, 24 h/d. Each behavioral response (i.e., a lick) was recorded with a 30 ms resolution (∼33 Hz sampling rate).

#### Habituation

Habituation is used to acclimate the mice to drinking from the BI waterspout without sounds being presented. Behavioral trials occurred at random intervals between 30 and 300 s. Water was released from the waterspout for 5 s during each trial. Habituation typically lasted 4 d.

#### Shaping

After the average lick rate for a cage showed sustained licking during habituation, they were moved on to shaping, in which the mice begin to associate hearing pure tones and licking the waterspout to receive water. Each trial was 4 s. The first second was silent, followed by a 1 s 11.3 kHz pure tone, and ending with 2 s of silence. Intertrial intervals (ITIs) were randomized between 5 and 9 s. The mice were required to refrain from licking for at least 5 s during the ITI to initiate the next trial. The first lick made during a 3 s behavioral response window after a tone onset (i.e., a hit response) was rewarded with water. On 20% of trials water was automatically released 0.5 s after the tone onset. Water was released on the remaining 80% of trials only after a behavioral hit. Shaping typically lasted 3–4 d.

#### Detection

Once the average lick rate began to increase after tone presentation, and once the lick latency distribution showed first licks regularly occurring after tone onset, we advanced the mice to a pure-tone detection task. The trial structure remained the same as in shaping; however, the freely available water on 20% of trials was removed and a response made during the 1 s silence before the tone (i.e., “early” responses) resulted in a punishment. If a mouse licked the waterspout during the 1 s silence, then the tone was still presented, but no water was delivered for subsequent licks, and a 20 s timeout was added to the ITI. The mice were also required to refrain from licking for at least 5–25 s during the ITI to initiate the next trial. The punishment of early licks and the 5–25 s wait period teach the mice to carefully control licking as to indicate tone detection. We also collected audiograms by randomizing the pure-tone frequency (4–45 kHz) and level (30–60 dB SPL).

#### Discrimination

We tested pure-tone frequency discrimination by training mice to lick in response to a target tone frequency (5 kHz) and to refrain from licking in response to a nontarget tone frequency (2 kHz). The mice were punished with a 20 s time-out for licking after the onset of a nontarget tone. All other task contingencies remained the same as in detection. This version of a go/no-go discrimination task functions as a stop-signal task, wherein the false alarm rate indicates the ability of the mouse to inhibit licking the waterspout in response to hearing the nontarget. An auditory reversal task could also be implemented using the ToneBox discrimination task. Reversal tasks are important for measuring cognitive flexibility. The user simply reverses the frequency parameters for the target and nontarget tones.

### Behavioral response rates

Early rates were calculated as the percentage of total trials in which the first lick was during the 1 s prestimulus silence. Hit and miss rates were calculated as the percentage of target tone trials with or without a first lick during the behavioral response window, respectively. False alarm and correct rejection rates were calculated as the percentage of nontarget tone trials with or without a first lick during the behavioral response window, respectively.

### Health monitoring

**Figure 2.**
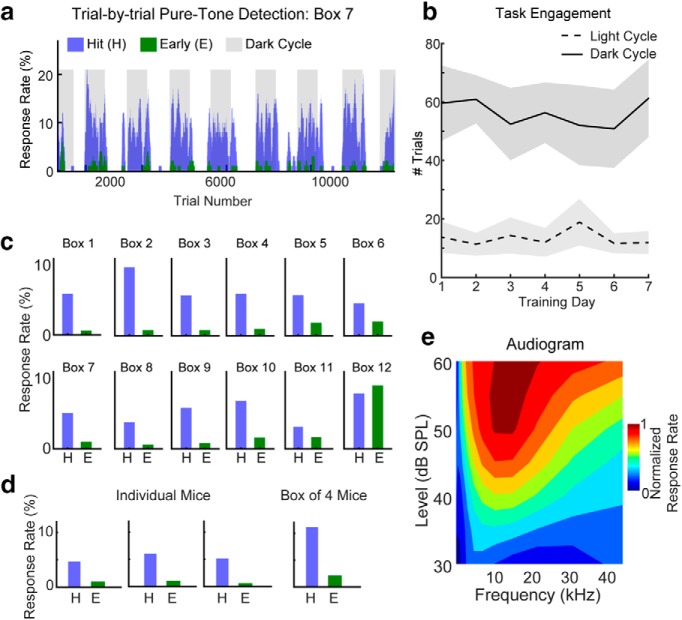
Auditory detection task performance in mice trained using ToneBoxes**. *a***, Example of behavioral data collected over 9 consecutive days in a single box of two mice. Response rates were calculated using a 25-trial sliding window. ***b***, Task engagement was defined as the average number of trials with at least one lick per trial. The solid and dotted lines show the dark and light cycles, respectively. The Shading shows ±2 SEM. ***c***, Hit and early response rates for each of the 12 tested boxes, color coded as in ***a*. *d***, Hit and early response rates for three individual mice that were initially trained in pairs, then isolated and trained alone (left three panels), and a box of four mice initially trained together (right). Data color coded as in ***a***. ***e***, Pure-tone detection with roving tone frequency and level. Hot and cool colors indicate high and low hit rates, respectively.

During detection and discrimination trials, the mice received 0.075 ml/hit. Given that a cage of two mice does ∼64 trials/d (Fig. 2*b*), each mouse received ∼2.4 ml of water/d, if divided evenly between the pair of mice in a cage. Since our system does not track individual mice, we cannot determine the exact distribution of water per mouse. We inspected the mice daily to ensure normal hydration by monitoring skin turgor, appearance, and weight loss. Skin turgor and appearance were rated daily on a scale of 1–5 as follows: (1) normal skin turgor and posture; (2) skin turgor present (<1 s); (3) hunched posture, piloerection, skin turgor 1–2 s; (4) eyes sunken, severe fuzzy facial fur, skin turgor up to 3 s; and (5) failure to right itself or grasp cage bars, acts paralyzed. Intervention to correct the health of the animal would occur after a rating of 3; however, we did not observe any rating >2 throughout the study. All mice were visually inspected every day and appeared to be healthy and active. To confirm the health of the mice, we weighed each mouse in each cage once a day for 5 d of detection trials after a few months of continuous training. The average weights (±SD) for each day were as follows: 30.3 ± 6.6, 30.5 ± 6.6, 30.9 ± 6.7, 31.0 ± 6.5, and 31.0 ± 6.5 g. The average weight over 5 d was 30.7 ± 6.0 g/mouse, which is typical for adult C57BL/6J mice. Since the mice remained healthy during our study, and task performance was the only means of getting water, we presume that all animals performed the task.

### Statistical comparisons

All statistical testing was done in Matlab software using a two-tailed nonparametric Kolmogorov–Smirnov (KS) test (*n* = 12 cages). The KS test is appropriate since we only sought nonparametric pairwise comparisons of empirical behavioral response distributions.

### Code accessibility

The code/software described in the article is freely available on-line at https://github.com/KanoldLab/ToneBox. The code is available as Extended Data 1. The results of this study were obtained using the Windows 10 operating system on a PC.

10.1523/ENEURO.0121-19.2019 Extended Data 1 ToneBox.zip contains a manual with parts lists and comprehensive step-by-step instructions for assembly and operation of the ToneBox system. We also provide models for 3D printing and PCB manufacturing, as well as software for system operation and data analysis. Download Extended Data 1, ZIP file.

## Results

**Figure 3.**
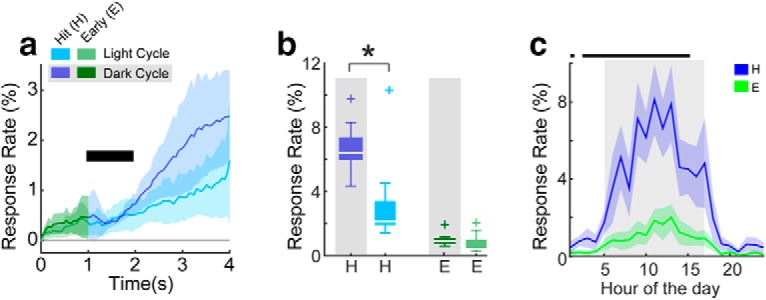
Statistical distributions of tone detection behavioral response rates across 12 ToneBoxes. ***a***, Behavioral response rate histograms across time in a trial for hits during the dark cycle (dark blue) and light cycle (light blue), and for early responses also during the dark cycle (dark green) and light cycle (light green). Shading shows ±2 SEMs. ***b***, Response rate-based task performance accuracy box plots. The star indicates that the dark cycle hit rate was significantly above the light cycle hit rate (*p* = 0.019, KS test; *n* = 12 cages). Data color coded as in ***a***. The + marks indicate data points outside of the 25th and 75th percentiles. ***c***, Hit and early rates shown for each hour of the day. The bin for each hour begins at tick marks. The dark cycle is shown in the shaded region. The thick black line shows when the hit rate was significantly above the early rate (*p* < 0.01, KS test; *n* = 12 cages). Shading shows ±2 SEMs.

**Figure 4.**
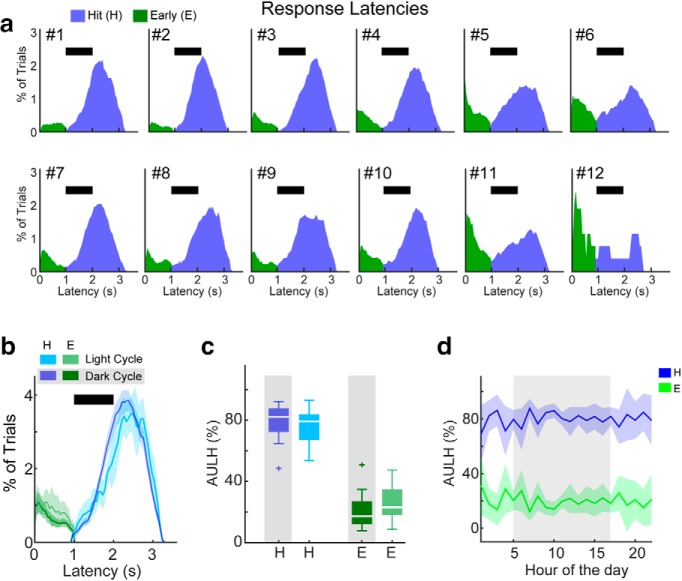
Pure-tone detection behavioral response latencies (i.e., the delay from trial onset until the first lick of the waterspout in each trial). ***a***, Latency histograms for each cage. The black bar shows when the 1 s tone was presented. ***b***, Average response latency distributions across 12 ToneBoxes. ***c***, Latency-based task performance accuracy box plots. Performance accuracy was defined here by the AULH (***a***) for hit and early responses. Shading shows ±2 SEMs. Data color coded as in ***b***. ***d***, Hit and early latency-based task performance accuracy shown for each hour of the day. The dark cycle is shown in the shaded region. Shading shows ±2 SEMs. Data color coded as in ***b***.

The ToneBox uses freely programmable sounds to train mice on operant conditioning tasks (Fig. 1). For our current use, we programmed the system to present pure tones (Materials and Methods). We used auditory tasks because the auditory system is crucial for human and animal communication, auditory deficits are associated with many sensory and cognitive diseases (Corcoran et al., 2002; Ewing and Grace, 2013; Zhou et al., 2015), and mice are readily trained on auditory tasks (Kurt and Ehret, 2010; Gess et al., 2011; Poddar et al., 2013; de Hoz and Nelken, 2014; Francis and Kanold, 2017). The ToneBox can easily be augmented to allow for different stimulus modalities using the additional general purpose digital output ports to control, for example, an LED. Each ToneBox is composed of a sound-attenuating box (≥20 dB attenuation between boxes for tones >1 kHz) that contains the home cage, a BI, and a CCU (Fig. 1*a–d*). The water delivery system routes a water line into the waterspout of each BI. The ToneBox was intentionally designed for scalability and use in animal facilities; thus, it is low cost, contains Wi-Fi connectivity, and allows easy integration with existing home cages. Thus, we set up 12 ToneBoxes in our animal housing facility (Fig. 1*e*). For initial experiments (Figs. 2*–*4), we placed two P30 C57BL/6J same-sex littermates into each ToneBox, totaling 24 mice (12 male, 12 female).

The ToneBox is operated with ToneControl software in Matlab. ToneControl uses a private wireless network to detect and interact with individual CCUs. A flat acoustic spectrum is critical for accurate testing of auditory function. We thus included automatic speaker calibration in ToneControl. Calibration only requires the user to have a microphone and an amplifier. We used Brüel & Kjær 4944A and 1704-A-001 equipment, but most ultrasonic amplifiers with an analog output will work. Ultrasonic calibration is possible up to ∼45 kHz using the specified BI and CCU components, and a sound card with at least a 96 kHz sampling rate.

A primary goal of automation is to acquire reliable results when testing large animal populations, which is, for example, required to identify potentially subtle effects of pharmacological, genetic, or environmental manipulations. Here we compared task performance during light versus dark cycles in the animal housing facility for both pure-tone detection and frequency discrimination tasks. In brief, during each trial of pure-tone detection, the trial began with a 1 s silence, followed by a 1 s tone, and then a 2 s silence. The intertrial interval was randomized between 5 and 9 s. If a mouse licked the waterspout after the target tone onset (i.e., a “hit” response), then water was delivered for 2 s. If the mouse licked during the 1 s silence before the tone (i.e., an early response), the target was still presented and licks were still recorded, but no water was delivered and the mouse was given a 20 s time-out. Between trials, the mouse was required to refrain from licking the waterspout for 5–25 s before the next trial began. The distinction between the ITI and the lick-refrain period was only that licking during the ITI per se did not affect when the next trial began. For pure-tone frequency discrimination tasks, either a target or a nontarget tone was presented with equal likelihood (i.e., 50%) in each trial. A lick after a nontarget (i.e., a “false alarm” response) was punished with a 20 s time-out, while all other task contingencies were the same as for target trials. The ToneBox also allows users to randomize tone levels across trials and include silent probe trials.

The user selects one of the following four training phases: habituation, shaping, detection, and discrimination. Here we focus on the core phases of detection and discrimination. Figures 2*–*4 show the results of training 12 cages of 24 mice on pure-tone detection tasks. The training session was run continuously for 9 d while the data were analyzed on-line with the provided ToneGraph software (Materials and Methods). Figure 2*a* shows an example of trial-by-trial response rates (i.e., hit and early rates) for a single cage. Since each trial of the task was time stamped, we were able to identify the circadian rhythm of the mouse through behavior, which is evidenced by the concentration of behavioral activity during the dark cycle of the room (Fig. 2*a*, gray). White overhead lights were kept on from 5:00 P.M. to 5:00 A.M. during the light cycle. The white lights were turned off, and red overhead lights were turned on from 5:00 A.M. to 5:00 P.M. during the dark cycle. The time stamping of trials also allowed us to quantify task engagement at different timescales across 7 full days of training (Fig. 2*b*). The average number of trials per circadian cycle with at least one lick per trial was greater during the dark versus light cycle (54.1 ± 10.5 vs 13.0 ± 3.3, *p* < 0.001). The average number of trials per minute per day was 0.07 ± 0.01. Our results for the light versus dark cycles indicate that the ToneBox system can be used to study how circadian rhythms affect performance across the day, without artificially disrupting the wakefulness of animals, as is done when removing animals from their home cage for manual behavioral training.

For each of the 12 boxes, we calculated hit and early response rates across the training period of 9 d. Figure 2*c* shows that hit rates tended to be greater than early rates for all boxes except box 12. These data indicate that the ToneBox is highly effective at automatically training large populations of mice to detect sounds.

Our initial experiments on tone detection used two mice per cage. However, some researchers may prefer to train individually housed mice or perhaps multiple cage-mates together. The latter scenario, together with the ease of ToneBox scalability, would allow for the simultaneous automated training of hundreds of mice per study. We find that individual mice (two males, one female) initially trained in pairs perform well when separated and continued training individually (Fig. 2*d*, three left columns). In addition, larger groups of four mice initially trained together in one home cage (four females) also collectively perform well on the task (Fig. 2*d*, right column). Thus, one need not train mice individually if one requires only a population of trained mice with a statistical distribution of expertise across cage mates. Individual mice from groups could be tested individually when needed. Given that other commercial behavioral systems are orders of magnitude more expensive than the ToneBox, our system remains scalable for individual or groups of mice. Our data show that mice learn to perform the tone detection task equally well when trained as individuals, pairs, and in larger groups.

Behavioral phenotyping may require multiple behavioral tasks to test the full range of sensation in a given modality. The ToneBox system allows different kinds of behaviors to be tested simultaneously in separate cages. For example, while most boxes continued to do the ongoing detection task with one pure tone at a fixed frequency and level, we stopped the training in a single box to allow for testing of auditory detection sensitivity by randomizing by tone level and frequency (Fig. 2*e*). Using this procedure, we are able to determine the behavioral audiograms of the mice. Hot colors in Figure 2*e* indicate better task performance, which was higher for more intense tones and best near 12 kHz, which is consistent with previous findings in healthy mice obtained using classic manual methods (Radziwon et al., 2009).

Our data suggest differences in task engagement across the light and dark cycles (Fig. 2*a*). To quantify how task engagement varied during light and dark cycles, we averaged tone detection response rates separately within the dark and light cycles (Fig. 3). In general, we found that hit rates were higher during dark versus light cycles (6.5 ± 1.0% vs 3.9 ± 1.8%, *p* = 0.017), and early rates were less than hit rates (dark: 2.08 ± 1.60%, *p* < 0.001; light: 1.06 ± 0.51%, *p* = 0.002; Fig. 3*b*). Early rates were not different during dark versus light cycles (*p* = 0.24). Figure 3*c* shows the average hit and early rates across each hour of the day. Response rates increased just before the onset of the dark cycle (Fig. 3*c*, gray region), peaked near the middle of the dark cycle, then decreased and stayed low for the remainder of the day. Hit rates were significantly above early rates both during the hours leading up to the dark cycle and during most of the dark cycle (*p* < 0.01). Hit rates peak between 9:00 A.M. and 1:00 P.M. Toward the end of the dark cycle, and during most of the following light cycle, hit rates and early rates were not significantly different. These analyses show that task engagement is not uniform across the light/dark cycle. Rather, there is an optimal time window for task engagement that peaks midway through the dark cycle.

Each behavioral response (i.e., a lick) is recorded with a 30 ms resolution (∼33 Hz sampling rate), which allows fine time analysis of behavioral responses to quantify behavioral accuracy. For example, Figure 4*a* shows histograms of behavioral response latencies (i.e., the delay from trial onset until the first lick of the waterspout for each cage in each trial). For 9 of 12 cages, the response latency functions peaked near the end of the pure tone (mean peak latency for all cages, 1.75 ± 0.59 s), indicating that most mice tended to lick only after the onset of the pure tone. We note that the response latencies may include a delay to approach the waterspout that depends on where the mouse was in the cage when it heard the tone.

Figure 4*b* shows the average response latency histograms for light versus dark cycles across cages. The functions appeared quite similar for light and dark cycles, suggesting that a response latency-based metric of task performance accuracy may be less influenced by overall behavioral activity than behavioral response rates. We thus quantified auditory detection accuracy by taking the percentage of the area under the latency histogram (AULH) for hit and early responses (Fig. 4*a*, blue and green curves, *c*, *d*). We found that the AULH was greater for hit versus early responses for both dark and light cycles (dark: 79 ± 7.4% vs 20.4 ± 7.3%, *p* < 0.001; light: 75.0 ± 6.7% vs 24.7 ± 6.6%, *p* < 0.001), indicating good task performance accuracy. Moreover, we found that task performance accuracy for dark versus light cycles was similar (hit_dark_ − hit_light_ = 4.0 ± 5.5%, *p* = 0.43; early_dark_ − early_light_ = −4.3 ± 5.4%, *p* > 0.05). Task performance accuracy might vary within the dark or light cycle. We thus reanalyzed the latency histograms in hourly time bins. Figure 4*d* shows the AULH analysis across each hour of the day. This analysis revealed that detection accuracy was consistently high across both light and dark cycles. This similarity in detection accuracy is in marked contrast to the light/dark cycle dependence of response rates (Fig. 3*c*). Together, our results indicate that most mice in cages were well trained (i.e., the mice usually refrained from licking unless the pure tone was detected). Significant increases and decreases in hit rates anticipated the change to dark and light cycles in the room, respectively. The overall lower hit rate during the light cycle compared with the dark cycle most likely reflects water satiation and that the mice were likely sleeping, as is expected from nocturnal animals (de Hoz and Nelken, 2014). Despite the circadian rhythmic modulation of overall behavioral activity (Fig. 3*c*), the latency of behavioral responses during pure-tone detection indicate that task performance accuracy was similar for dark and light cycles (Fig. 4*d*). Thus, the performance in an auditory detection task is independent of the circadian rhythm.

**Figure 5.**
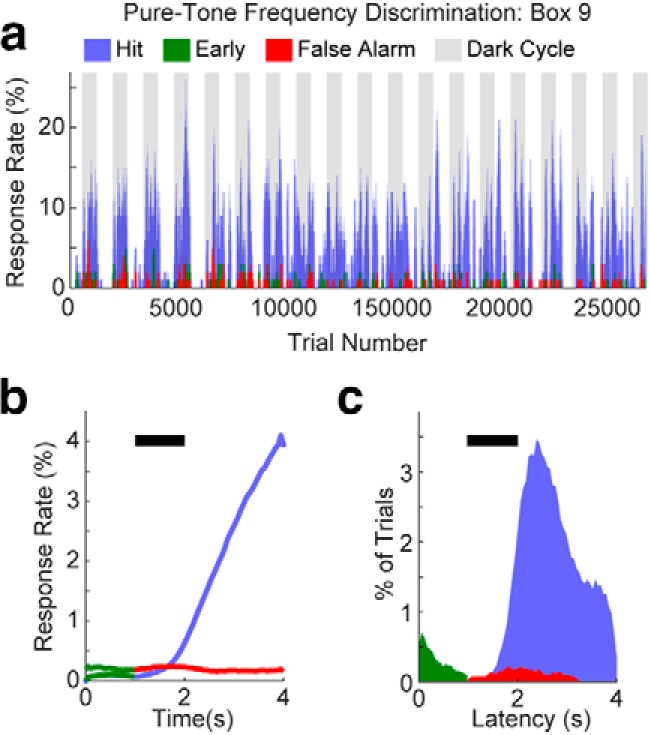
Pure-tone frequency discrimination task performance. ***a***, Example of behavioral data collected over 19 consecutive days in a single ToneBox of two mice. ***b***, Hit, early, and false alarm response rate histograms across time in a trial, color coded as in ***a*. *c***, Hit, early, and false alarm latency histograms across time in a trial, color coded as in ***a***.

**Figure 6.**
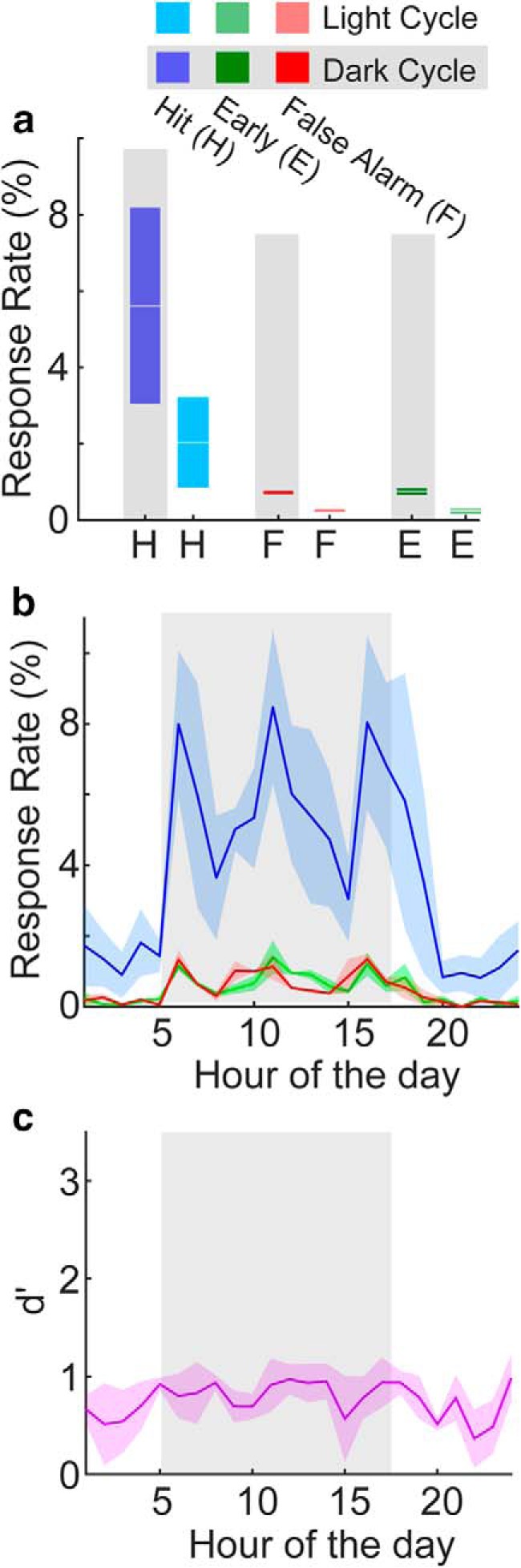
Statistical distributions of tone discrimination response rates across two ToneBoxes. ***a***, Response rate-based task performance accuracy box plots for hits during the dark cycle (dark blue) and light cycle (light blue), false alarms during the dark cycle (dark red) and light cycle (light red), and for early responses also during the dark cycle (dark green) and light cycle (light green). ***b***, Hit, false alarm, and early rates shown for each hour of the day. The dark cycle is shown in the shaded region. Data color coded as in ***a***. Shading shows 2 SEMs. ***c***, Response rate-based *d*´ values for each hour of the day. Shading shows ±2 SEMs.

**Figure 7.**
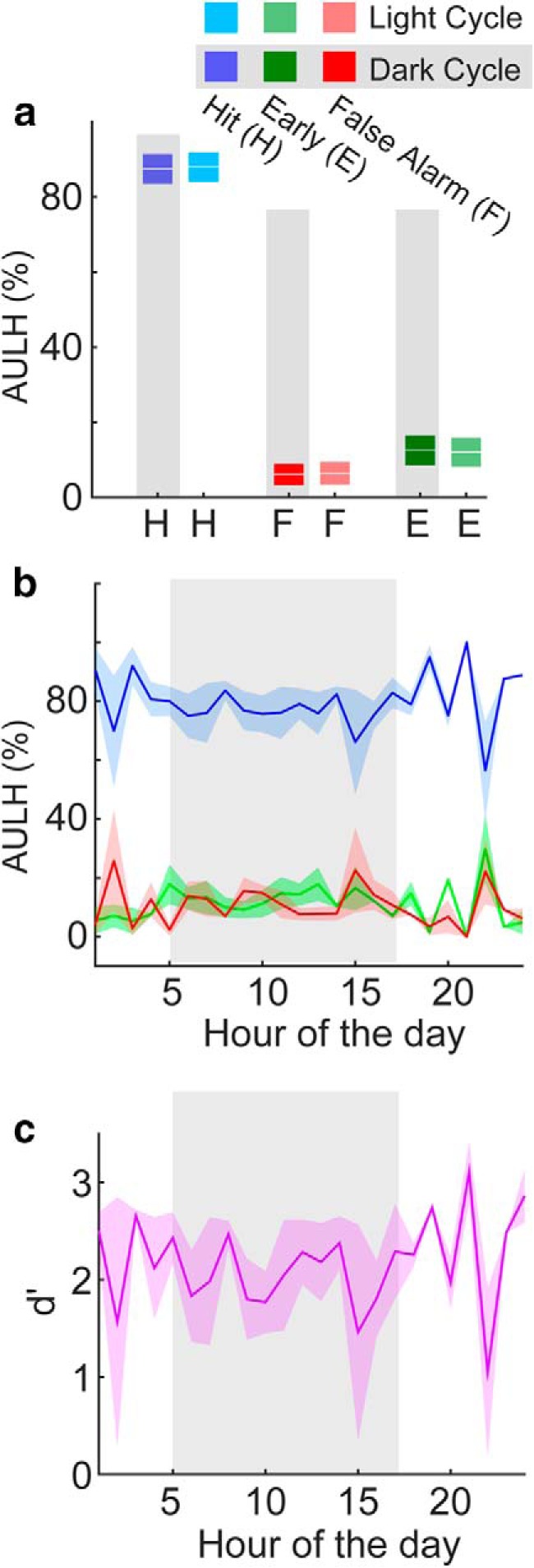
Statistical distributions of tone discrimination response latencies across two ToneBoxes. ***a***, Latency-based task performance accuracy box plots. Performance accuracy was defined here by the AULH for hit and early responses. ***b***, Response latency-based task performance accuracy shown for each hour of the day, and for hit, false alarm, and early responses. The dark cycle is shown in the shaded region. Shading shows ±2 SEMs. Data color coded as in ***a***. ***c***, Response latency-based *d*´ values for each hour of the day. Shading shows ±2 SEMs.

We reasoned that task difficulty might affect task engagement. Thus, having established a behavioral baseline for auditory detection, we sought to study how increasing task difficulty affected behavioral activity. We thus trained mice in two ToneBoxes on a pure-tone frequency discrimination task (Figs. 5–7). The discrimination task is more difficult than tone detection because the mouse must classify tones as a target versus nontarget before making a behavioral choice—which requires a behavioral inhibition that is absent in the detection task. Figure 5*a* shows an example of trial-by-trial behavioral activity for hit, early, and false alarm trials across 19 continuous days of training for an individual box of two mice. As in tone detection, most behavioral activity was concentrated during the dark cycle for tone discrimination, and most responses were hits. Figure 5, *b* and *c*, shows the response rates and latency histograms for this cage. Both the response rate and latency functions peak after the tone onset, indicating that the mice were able to discriminate target versus nontarget tones. Figure 6*a* shows the average response rates for the two cages—confirming that the mice were more active during the dark cycle.

A comparison of the results from the detection task (Fig. 3*c*) with those of the discrimination task (Fig. 6*b*) revealed task-dependent differences in the hourly response rates. Only for the discrimination task was there a periodic modulation in both tested cages, with response rates repeatedly rising and falling approximately every 3–4 h during the dark cycle. These data suggest that when task difficulty increases, the mice tend to take periodic breaks in task performance. However, it is possible that the periodic modulation may be an effect of continued training on a go/no-go task, rather than due to task difficulty per se.

We next investigated whether there were variations in discrimination task accuracy across the dark cycle. The discrimination task provides both hit and false-alarm trials, and we thus computed the discrimination sensitivity (*d´*) values to quantify discrimination sensitivity (Fig. 6*c*). In contrast to the response rates per se (Fig. 6*b*), we found that the *d*´ value was generally consistent across the circadian cycle. This hourly *d*´ analysis of the discrimination task supports our finding from our analysis of response latency: once engaged with the task, the mice show consistent performance accuracy throughout the circadian cycle. Moreover, AULH analysis of hit, early, and false alarm responses during the discrimination task (Fig. 7) also show that task performance accuracy was consistently high for both light and dark cycles. Thus, while task difficulty can cause variations in task engagement during the dark cycle, task accuracy remains constant.

Here we demonstrate the ToneBox for use in tone detection and tone discrimination tasks. In addition to testing basic sensation, frequency discrimination as implemented here can be used to test cognitive flexibility and impulsivity in a reversal or stop-signal task (Logan et al., 2014), respectively (Materials and Methods).

## Discussion

These results show that the ToneBox system can simultaneously train many mice on different tasks and can thus be used to efficiently phenotype animal behavior. Benefits of the ToneBox system include that (1) it fits within existing commercial home cages—which reduces cost and eliminates the need for additional laboratory space to train mice; (2) it is open-source, and thus fully customizable; (3) it can produce ultrasonic sounds and provides automated acoustic calibration; and (4) it is cost effective—a single ToneBox costs under $250—making it a truly scalable system. While the current design of the ToneBox does not track the behavior of individual mice, the Raspberry Pi-based design allows knowledgeable users to add, for example, cameras or RFIDs (radio-frequency identification), to track individual mice. As a low-cost and high-throughput tool designed for both entry-level and expert behaviorists, we expect the ToneBox to be a transformative tool in behavioral phenotyping and drug discovery, and for understanding the neural basis of perception, cognition, and action. When combined with neuroimaging in freely behaving mice, the ToneBox has the potential to facilitate rapid new discoveries in neuroscience research.

## Synthesis

Reviewing Editor: Darcy Kelley, Columbia University Decisions are customarily a result of the Reviewing Editor and the peer reviewers coming together and discussing their recommendations until a consensus is reached. When revisions are invited, a fact-based synthesis statement explaining their decision and outlining what is needed to prepare a revision will be listed below. The following reviewer(s) agreed to reveal their identity: Livia de Hoz.

While the reviewers are in agreement that the automated behavioral testing unit (ToneBox) is a potentially valuable addition to currently available technologies, particularly for auditory research, a number of major concerns were raised in the reviews with respect to experimental rationale, clarity of procedures, measurements, representation of data and statistical that prevent informed evaluation and must be addressed.

Some examples of major concerns:

1. The ToneBox URL was not available to for review as a result of the double blind process.

2. Extended Testing: The procedure has 4 components: Habituation (4d), Shaping (3-4 d), Detection (no stated duration; data from 9 dark/light cycles presented) and Discrimination (no stated duration n, data from 19 dark/light cycles presented). Food was available ad libitum. Mice were water deprived for 23h prior to Habituation. While water is described as available “ad libitum” during Habituation (line 108), availability is also described as limited to the first 5s of each 10s trial (line 113). The duty cycle us described as “1h”; again not “ad libitum”. Water was restricted to “hits” (task defined successful licks) during Detection and Discrimination trials. How much water was dispensed per trial and how was it determined whether each member of the pair received sufficient water across the tasks as the tasks were not self-initiated. The BI produces a tone and the animal must lick or not lick (according to the rules of the task) within some window of time. To test the animals' engagement and accuracy during different phases of their circadian rhythm trials must be presented at all hours of the day. Does the presentation of sounds during the animals sleep phase in anyway disrupt their natural sleep/wake cycle? Did the extended testing affect animal's physiological state (weight, for example) in a way that might impact animal welfare? Were cages checked daily during (presumably) cleaning?

3. Test design: Because the Discrimination task was run last, attribution of the modulation of licking rhythms observed (“breaks”) to cognitive difficulty of the task is confounded with testing order (and possibly changes in physiological state). The sounds are presented during both light and dark cycles. During detection, the circadian rhythm is maintained suggesting that sound presentation, even at high intensities, does not affect sleep. However, during the later days of discrimination learning the circadian rhythm appears to be affected, again suggesting a change in physiological state (see above).

4. Rationale for testing paradigm: What explicitly are the “cons” as well as the “pros” of the behavioral interface design? Group testing (especially in these behavioral units where the animals live for days at a time) against single animal analysis? Could an imaging technique such as depth sensing be added or animals individually tagged in some way? Are all animals in the box performing the task? See comment about response rates. This concern plays out, for example, in the reported mean peak latency of the discrimination response to auditory stimuli for all cages of 1.75s +/- 0.59s. Since the trials are not self-initiated, the animal could be on the other side of the cage when the tone is played. Without video tracking, it's not possible to know where the animal is when the tone is played and therefore how much of the delay is a true response latency versus a motor delay to get to the lick spout. Do cage mates ever compete for the opportunity to lick which would also affect latency?

5. A more complete description of the benefits and drawbacks of the Behavioral Interface unit is required. For example, the BI does not allow identification of individual animals tested in the same box. Can this feature be added via tagging? It is not clear whether cage mates ever interfere with each other while at the BI. The authors state that the mean peak latency for all cages during Discrimination testing is 1.75s +/- 0.59s; the response seems slower and more variable than might be expected. Without video tracking, it's not possible to know where the animal is when the tone is played and therefore how much of the delay is a true response latency versus a a motor or competition to reach the lick spout.

6. Subjects: Twelve same sex pairs of 30d old mice as well as one cage of 4 mice (sex?) were trained and tested. With this small N, is not possible to determine if the BI approach is actually scalable. Three mice were trained and tested individually. What sex were these mice; were they re-trained (trained again after initial group training) or trained before group training? Were the 4 individuals trained together in addition the 12 pairs? It is not clear whether cage mates ever interfere with each other while at the BI. Individuals of all species learn at different rates, even siblings can achieve significantly difference performance levels. Both individuals in box 12 in this study fail to adequately learn the task, but how what about individuals in other boxes? Are they both mediocre, or is one near perfect and the other poor?

7. Testing procedures: exactly how was the audiogram in Figure 2d obtained and used? What speaker was used? Were corrections applied using the B&K microphone? Were roving frequency and intensity tested for each trial? The procedure is opaque.

8. Measurements: Response rate is a major dependent variable (figures2,3,5 and 6) but no description of exactly how this is calculated is provided. Because only licks were recorded, there is no way of determining which mouse is providing this information or of assessing any differences across the 12 boxes of pairs, individual mice or the group of 4 mice. It is not clear whether testing pairs actually confers an advantage over individuals. Individuals of all species learn at different rates, even siblings can achieve significantly difference performance levels. Both individuals in box 12 in this study fail to adequately learn the task, but how what about individuals in other boxes? Are they both mediocre, or is one near perfect and the other poor? While the ability to separate individuals might not be necessary for certain experiments, this must be discussed.

9. Statistics: There is a general lack of details regarding how values are calculated and the statistical tests applied. The KS test was used for all comparisons however that test is only appropriate when determining whether the difference between two cumulative distributions is significant.

Detailed comments:

Reviewer A:

Major comments:

1. This unit does not allow distinguishing between individual animals tested in the same box reducing the benefits of group testing (especially in these behavioral units the animals live for days at a time) Could individuals be followed through tags or video?

2. Does ‘response rate (%)’ means different things in different plots ? Please clarify the meaning of this measure and consider using absolute numbers, not percentage. Please also plot the number of trials/minute/day so that the reader can get a feeling for how often the animals lick in between trials at the beginning.

3. Does the number of animals per box reiaibly affect this measurement? A measure of the number of trials in which at least one lick occurred, in blocks of, for example, 10 minutes during the 5 second window after tone onset, compared to 5 seconds before tone onset would be useful.

4. The authors say that the mice perform more ‘breaks’ when the task becomes more difficult. Are the animals performing at least one lick/trial on ‘go’ trials at a rate comparable to that of the detection phase (normalized to the number of go/detection trials)? Or are they licking in a smaller proportion of go trials but more often (i.e more licks per trial)?

5. Performance should be measured at a sampling rate smaller than 1 hour (perhaps10 minutes (ca. 30 trials?). This analysis should be performed individually for each box, since the break time can happen at different times.

6. Why did the number of hits increase during the light cycle at the end of the discrimination phase?

7. What evidence indicates that all animals in the box are performing the task?

8. It would be useful to have an estimate of the cost of each box (and the microphone if required).

Minor comments:

Line 5: number of mice?

Line 8: iIntroducing the concept of difficulty and breaks as they might relate to modulation.

Line 37: typo ‘writ large’

Sampling rate for the sound presentation (line 75) and the behavioral sensors?

Line 110: “for each 10s trial...” the reader cannot not know what this refers to at this stage.. clarify

Line 118: What tones were used during shaping?

Line 120: there is a typo in the brackets (it is) should be (ITIs)

Line 122: I.e. should be i.e.

Line 122: I assume in the remaining 80% of trials water is delivered only after a lick?

In the habituation and shaping, is the sound presented across ITIs 24 hours/day?

Line 161: I would add a reference to Ehret's work since he was the first to develop auditory tasks for mice.

. Line 175: B&K microphones are extremely expensive

Line 182-188: I think the description of the detection and discrimination are mixed. The ITI is 5-9s but the animal must refrain from licking for 5-25s?

Line 188 and equivalent section in methods: what is the proportion of target and non-target trials?

Do the trials occur throughout a 24 hour period?

Can individual animals be identified? Or is the combined behavior of the 2 animals in a cage reported?

Line 201. What does “trials done per day” mean? Are these trials in which the animal's perform a lick? Please clarify. Also, it should probably say ‘per phase’, but definitely not ‘per day’.

Line 218 and figure 2c. I would expect the response rate to be quadruple for the cage with 4 mice compared to the cage with single mouse. Why is this not the case?

Figure 2a. How are response rates (%) calculated? Is it the percentage of trials in which at least a lick was made (hit) out of all trials in a specific time window? Please clarify in text and figure legend.

Figure 2c. Why is the percentage of hits and early responses higher in the 4 animal cage than in the other cages?

Line 220. The sentence starting In line 220 doesn't make sense.

Figure 3a. What is the response rate (%) measuring in figure 3a?

Figure 3c. It looks like the thick bar representing significant is not in the right place. Also, it would make more sense to put the real hours of the day on the x axis, unless the dark phase really started at 5am? If this is the case, this should be stated in the methods section/animals.

Figure 4A, latency axis is not clear. Why not use exactly the same arrangement as in figure 5C?

Please clarify that it is the latency to the first lick in the 1st or 2nd 1- second window. with 30ms resolution in the figure legend.

Figure 5b. This measurement cannot be done per hour, it needs to have a finer resolution. I would do it per box in a resolution of 10 min windows and the average across boxes...

Reviewer B

1.A clarification for how hit rates are calculated and exactly what constitutes a hit is necessary. Is the first lick after the tone begins, no matter how long after, a hit, or are only licks in a defined time window considered hits? Is the hit rate then the percent of trials the animal licks following the tone? Comparing “rates” for example on lines 230-231 should not be done with a KS test. Also should not equivalent time windows be used for early and hits? The early window appears to only be 1s while the hit window is typically more than 2s.

3. On line 233 the authors state “Response rates increased just before the onset of the dark cycle” referencing figure 3c. This statement should be statistically justified. Looking at figure 3c the difference between hit and early is said to be significant at hours 0, 2, 3, and 4 but not at 16 or 17. odd given the error bars. Data are binned by hour but how? Is data at point 5 the data between 5 and 6 or between 4.5 and 5.5?

4. On line 236 the authors identify a “four hour window of peak wakefulness.” This statement should be statistically justified. Are those hours significantly higher than the one immediately before and after?

5. Throughout the paper the authors describe hit rate and early rates and only later include plots of d'. d' can be included in these early analysis since all 4 conditions are met hit: lick after tone, miss: no lick after tone, false alarm: lick during silence, correct rejection: no lick during silence. Plots of how d' changes across training days would be useful.

6. Starting on line 247 thee histogram for first lick time after the tone onset is described. The area under those histograms is then compared between the dark and light cycle and described an ROC analysis. Exactly how these data were analyzed must be explained. ROC curves are receiver-operator characteristic curves comparing true hits against false positives at different thresholds. Area under a histogram plot is not an ROC curve.

7. Figure 2a plots response rate as a function of trial number. How is this computed? Are trials binned? If so how?

8. Figure 2b which boxes show a significant difference?

9. Figure 3a (and all other figures) the n is never stated in results or statistical comparisons. Is the n using boxes, mice, days, trials...?

10. Figure 3B the error bars described on line 395 as “2 standard errors of the mean (SEM)”. However, data are usually plotted with errors bars that represent the SEM or some multiple of standard deviations, e.g. 2 SD, or some measure of confidence, e.g. 95% confidence interval. Since SEM depends strongly on the n this must be reported.

11. Figure 4a: why do all histograms fall to 0 by the same value ~3.2s? Since the mouse cannot know exactly when the tone has started playing, taking into account delay in perception, one would expect early responses to bleed into the tone slightly. Also most early responses appear to decrease in frequency as the tone onset approaches. There is a sharp discontinuity at exactly 0s. Since the animal cannot know when the tone is going to play how do the authors explain this drop? The histogram for box 12 appears very blocky which might be expected from a low n (not reported) but given the data from figure 1 one would expect an n equivalent to the other boxes. A clarification of how these plots are made, what data is included and excluded is required.

12. In Figure 4b, what trials are included? Is it only trials where the animal makes a response? Why this plot falls to 0 at ~3.2s while the plot in Figure 3a continues to rise at 4s is confusing. And what do the + marks represent?

13. Figure 4c If the authors are only analyzing trials where the animal makes a response then the AULH must add to 1. Separating hits from early is redundant, as early must equal 1 - hit, and one can simply plot the ratio. As discussed earlier d' might be a better measure of performance in this regard.

14. Figure 5 would benefit from a plot of d' as a function of day to show more clearly how learning and performance improve.

15. In Figure 6a the data for early responses and more especially false alarms seems to have extremely low variability. Do all samples have almost exactly the same rates?

16. Figure 6b is the modulation during the dark cycle significant? Is the modulation seen in both boxes?

Minor Concerns:

Typo line 120 “(it is)” should be “(ITIs)”.

Typo line 122 “(I.e.” should be “(i.e.”.

The speaker in the BI is said on line 176 to be able to produce tones up to ~45 kHz. What speaker was used to generate ultrasonic sounds reliably.

On line 183 “The inter-trial interval was randomized between 5-9 s.”, but on line 187 “the mouse was required to refrain from licking the waterspout for 5-25 s before the next trial will begin”. Are these durations for different stages in the training? If so please make that explicit, if not please reconcile this discrepancy.

Adding values on line 202 of trials done per day yields ~55 which seems quite low. How does this value compare to other training setups where values in the many hundreds are more typical. Is this value per animal or per cage?

On line 213: “mice trained in groups perform well when tested individually (Figure 2c”, but in the figure legend on line 385 “c. Hit and early response rates for 3 individual mice that were isolated from a group and trained alone”. Which is correct? Clarify.

The sentence beginning with “For example,” on line 220 does not make sense as worded.

Values for error should be reported to the same number of decimal points as the main value; line 230 should read “6.5% +/- 1.0%”.

line 236 the authors hours should be given as time relative to the dark/light cycle period.
