# Supplementary material for: Automated Behavioral Experiments in Mice Reveal Periodic Cycles of Task Engagement within Circadian Rhythms
Source: eNeuro. 2019 Sep 17;6(5):ENEURO.0121-19.2019. doi: 10.1523/ENEURO.0121-19.2019 (PMC6775758; doi:10.1523/ENEURO.0121-19.2019)
Supplement: Extended Data 1 — ToneBox.zip contains a manual with parts lists and comprehensive step-by-step instructions for assembly and operation of the ToneBox system. We also provide models for 3D printing and PCB manufacturing, as well as software for system operation and data analysis. Download Extended Data 1, ZIP file. [file sup_enu-eN-MNT-0121-19-s02.zip › ToneBox-master/ToneBox_Manual.docx]

**ToneBox v1.0**

**
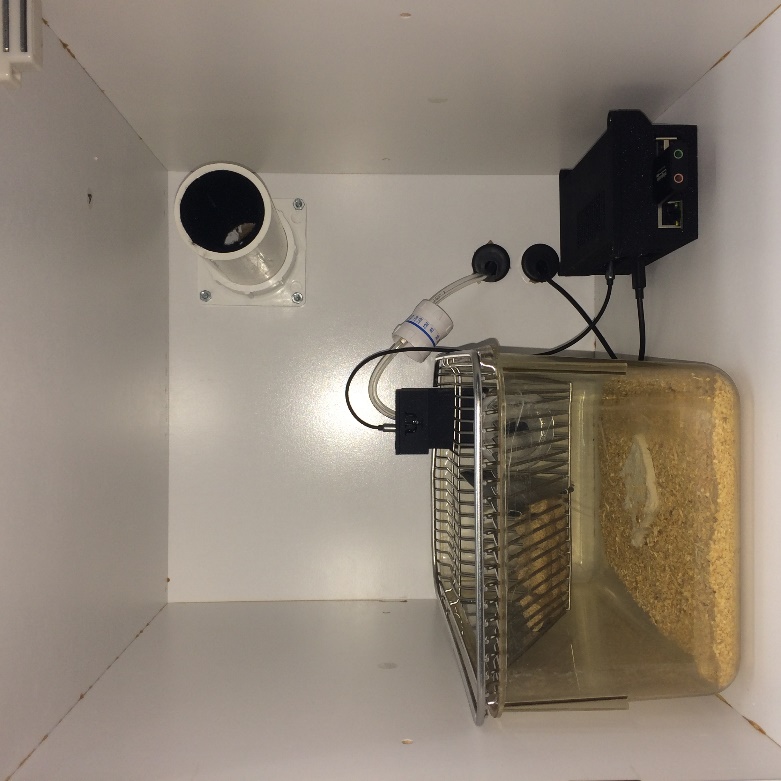
**

Nikolas A. Francis, Kayla Bohlke, Mena Mohamed, Patrick Kanold

University of Maryland

2019

**
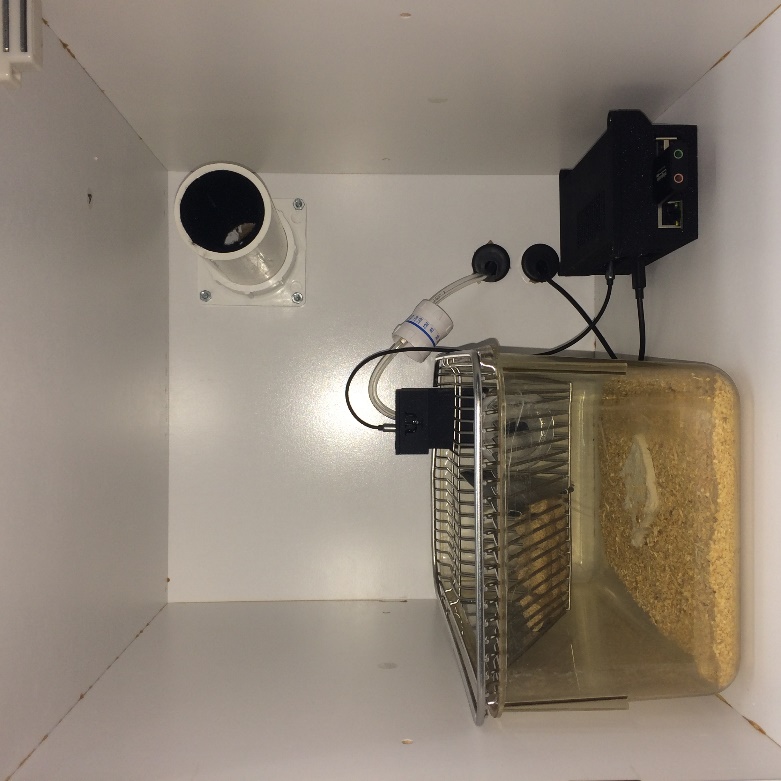

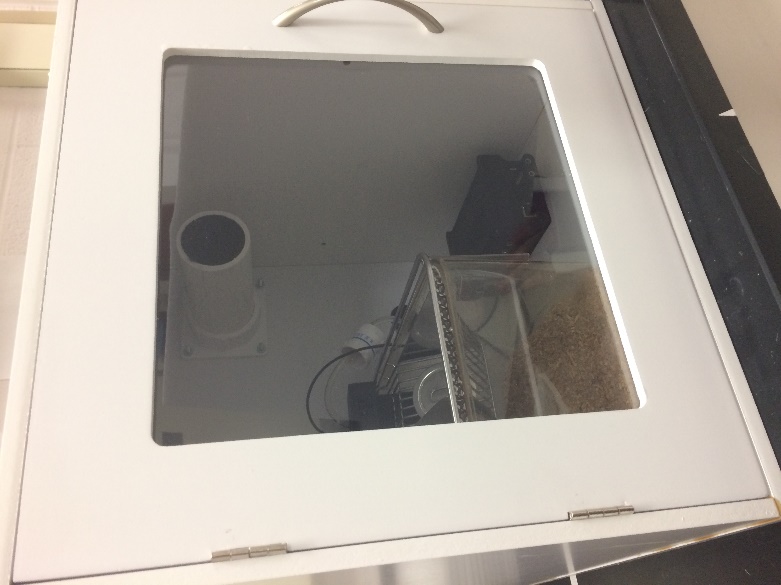
**The ToneBox trains mice on auditory operant conditioning tasks using a WiFi-connected Raspberry Pi to play sound and monitor behavioral responses. The ToneBox is operated using Matlab software. This manual describes the materials, assembly, and operation of the ToneBox.

**Materials**

This section describes the parts used for assembly. The ToneBox is composed of a Central Control Unit that houses the Raspberry Pi, a Behavioral Interface for the mouse cage, a system for water delivery, and a sound attenuating box. ‘#x’ refers to the number of items required for one ToneBox. Many of the small items come in large quantities that will supply multiple ToneBoxes.

*Assembly Tools*

1. [Soldering Iron](https://www.amazon.com/Weller-WLC100-40-Watt-Soldering-Station/dp/B000AS28UC/ref=sr_1_4?s=hi&rps=1&ie=UTF8&qid=1522249377&sr=1-4&keywords=soldering+iron&refinements=p_85%3A2470955011)
2. [Solder iron tips](https://www.amazon.com/Weller-WLC100-SP40L-Fine-tools/dp/B0747R26S8/ref=pd_sim_469_3?_encoding=UTF8&pd_rd_i=B0747R26S8&pd_rd_r=99H4WH10AVVMQGF4APFZ&pd_rd_w=WIy7h&pd_rd_wg=C0ay8&psc=1&refRID=99H4WH10AVVMQGF4APFZ)
3. [Soldering tip cleaner](https://www.amazon.com/Hakko-599B-02-Wire-type-soldering-cleaner/dp/B00FZPGDLA/ref=pd_bxgy_469_img_3?_encoding=UTF8&pd_rd_i=B00FZPGDLA&pd_rd_r=Q7XKEM05W1SKC4H6A17Y&pd_rd_w=WZMdq&pd_rd_wg=SHIyn&psc=1&refRID=Q7XKEM05W1SKC4H6A17Y)
4. [Solder](https://www.amazon.com/WYCTIN-0-8mm-Rosin-Soldering-Solder/dp/B071JS8Y53/ref=sr_1_1_sspa?ie=UTF8&keywords=solder%20wire&psc=1&qid=1516641028&refinements=p_85%3A2470955011&rps=1&s=hi&sr=1-1-spons)
5. [Philips screw driver](https://www.amazon.com/Craftsman-9-47139-Phillips-Screwdriver-Piece/dp/B002L0LIBM/ref=sr_1_3_sspa?s=hi&ie=UTF8&qid=1522335083&sr=1-3-spons&keywords=phillips+screwdriver+set+%22%231%22&psc=1&smid=AUU1KYNERU7US)
6. [Wire strippers](https://www.amazon.com/Hakko-CSP-30-1-Stripper-Maximum-Capacity/dp/B00FZPHMUG/ref=pd_sim_328_6?_encoding=UTF8&pd_rd_i=B00FZPHMUG&pd_rd_r=P7SZR6555Z386WD3F9PA&pd_rd_w=QiLAk&pd_rd_wg=Ie28d&psc=1&refRID=P7SZR6555Z386WD3F9PA)
7. [Wire cutters](https://www.amazon.com/Hakko-CHP-170-Micro-Cutter/dp/B0765NMV68/ref=sr_1_4?s=hi&ie=UTF8&qid=1522335428&sr=1-4&keywords=wire%2Bcutters&th=1)
8. [Dremel](https://www.amazon.com/Dremel-3000-1-25-Attachment-Accessories/dp/B00LUJONH6/ref=sr_1_4?s=hi&ie=UTF8&qid=1536430563&sr=1-4&keywords=dremel)
9. [Drill](https://www.amazon.com/BLACK-DECKER-DR260C-Drill-Driver/dp/B00T2VJ93C/ref=sr_1_11?s=hi&rps=1&ie=UTF8&qid=1536430639&sr=1-11&keywords=drill&refinements=p_85%3A2470955011)
10. [Stepped drill bit](https://www.amazon.com/Neiko-10197A-Titanium-Drill-5-Piece/dp/B002GQ5AKG/ref=sr_1_3_sspa?s=hi&ie=UTF8&qid=1536430671&sr=1-3-spons&keywords=Stepped+drill+bit&psc=1)

*Desktop Computer*

1. Windows 10 PC with WiFi
2. Matlab 2017b or later version
3. ToneControl software (see installation folder)
4. [WiFi router](https://www.amazon.com/TP-Link-Wireless-Portable-Travel-Router/dp/B00TQEX8BO/ref=sr_1_6?s=electronics&ie=UTF8&qid=1536431189&sr=1-6&keywords=mini+wifi+router)

*Central Control Unit (CCU)*

1. 3D printed case (see file CCU_Case.obj in installation folder)
2. [Raspberry Pi 3](https://www.amazon.com/Raspberry-Pi-RASPBERRYPI3-MODB-1GB-Model-Motherboard/dp/B01CD5VC92/ref=sr_1_3?s=electronics&ie=UTF8&qid=1522336492&sr=1-3&keywords=raspberry+pi+3)
3. [SD card](https://www.amazon.com/Samsung-MicroSD-Adapter-MB-ME32GA-AM/dp/B06XWN9Q99/ref=pd_bxgy_147_img_2?_encoding=UTF8&pd_rd_i=B06XWN9Q99&pd_rd_r=TG4GMGCFEDVHVVE4AC8Z&pd_rd_w=NCKkn&pd_rd_wg=Bya7R&psc=1&refRID=TG4GMGCFEDVHVVE4AC8Z)
4. 2x [Standalone Momentary Capacitive Touch Sensor Breakout - AT42QT1010](https://www.adafruit.com/products/1374)
5. CCU PCB from [Fritzing](https://aisler.net/fritzing) (see CCU_PCB.fzz file in installation folder)
6. [5.5mmx2.1mm 3Pins PCB Mounting Female DC Power Jack](http://www.amazon.com/gp/product/B00MJVIFS2/ref=pd_lpo_sbs_dp_ss_2?pf_rd_p=1944687742&pf_rd_s=lpo-top-stripe-1&pf_rd_t=201&pf_rd_i=B00LHKYDXM&pf_rd_m=ATVPDKIKX0DER&pf_rd_r=1HHJ4RCJ82E2YJVT77VY)
7. 7x [3 Pin PCB Mount Female 3.5mm Stereo Jack Socket Connector](http://www.amazon.com/Mount-Female-Stereo-Socket-Connector/dp/B008SNZUYC/ref=sr_1_1?s=hi&ie=UTF8&qid=1448908642&sr=1-1&keywords=10+Pcs+3+Pin+PCB+Mount+Female+3.5mm+Stereo+Jack+Socket+Connector&refinements=p_85%3A2470955011)
8. OPTIONAL: 2x Resistors (various ohms for digital output)
9. 1x [4.7 uF Capacitors](http://www.digikey.com/product-search/en?keywords=493-12781-1-ND)
10. 2x [22 uF Capacitors](https://www.digikey.com/product-detail/en/nichicon/UVR1E220MDD6TP/493-12773-1-ND/4328366)
11. 2x [Omron G5V-2-H1 DC3 RELAY GENERAL PURPOSE DPDT 1A 3V](https://www.digikey.com/products/en?keywords=G5V-2-H1%20DC3)
12. [Switch](https://www.digikey.com/product-detail/en/e-switch/100SP1T2B4M6QE/EG2362-ND/378831)
13. 1x [VCC 5302H1-5V Red PCB LED, T-1 3/4 RA, 8 mcd, Built-In Resistor, 5V, Red Lens](http://www.alliedelec.com/vcc-visual-communications-company-5302h1-5v/70130277/)
14. 2x [Break-away 0.1" 36-pin strip male header (10 pieces)](https://www.adafruit.com/products/392)
15. [PREMIUM FEMALE/FEMALE JUMPER WIRES - 20 X 3" (75MM)](https://www.adafruit.com/products/1951)
16. [FEMALE HEADER SOCKET 40 PIN](https://www.amazon.com/uxcell-2-54mm-40-Pin-Female-Connector/dp/B00R1LLM1M/ref=sr_1_9?ie=UTF8&keywords=FEMALE%20HEADER%20SOCKET%2040%20PIN&qid=1519845204&sr=8-9)
17. [BNC connector](https://www.sparkfun.com/products/10550)
18. 2x [Diode](https://www.digikey.com/products/en?keywords=%201N4001-TPMSCT-ND)
19. [12 V power w/ splitter](https://www.amazon.com/ANVISION-Adapter-Splitter-5-5x2-1mm-Efficiency/dp/B01C028BSS/ref=sr_1_2?ie=UTF8&keywords=12%20v%20splitter%206-way&qid=1499896782&s=electronics&sr=1-2) (1 per 4 boxes)
20. [Power extension cord](https://www.amazon.com/Hanvex-HDCQ3-Extension-Cable-Adapter/dp/B00FTGH38W/ref=sr_1_3?s=electronics&rps=1&ie=UTF8&qid=1515780064&sr=1-3&keywords=2.1+mm+extension&refinements=p_85%3A2470955011)
21. [1’ audio cable](https://www.monoprice.com/product?p_id=18629)
22. 2x [3’ audio cable](https://www.monoprice.com/product?p_id=18629)
23. [12’ audio cable](https://www.monoprice.com/product?p_id=18629)
24. [USB cable](https://www.amazon.com/Anker-PowerLine-Micro-USB-Smartphones/dp/B012VZ7MUM/ref=sr_1_3?s=electronics&rps=1&ie=UTF8&qid=1522258141&sr=1-3&keywords=micro+usb+cable+6%27&refinements=p_85%3A2470955011)
25. [USB sound card](https://www.amazon.com/Flujo-External-Headphone-Microphone-Jacks-Compatibility/dp/B078K6TT5F/ref=sr_1_3?ie=UTF8&qid=1549047803&sr=8-3&keywords=flujo+external+usb)

*Behavioral Interface*

1. 3D printed case (see file BI_Case.obj in installation folder)
2. 3D printed stainless steel waterspout (see file Waterspout.obj in installation folder)
3. 3x [3.5mm Stereo Jack Panel Mount Connector](https://www.amazon.com/gp/product/B01DBOBRHQ?redirect=true&ref_=ox_sc_act_title_7&smid=ABK5NCUPQ578S&th=1)
4. [Mono 2.5W Class D Audio Amplifier - PAM8302](https://www.adafruit.com/products/2130?gclid=Cj0KEQiAoby1BRDA-fPXtITt3f0BEiQAPCkqQWqv-4bG627WQL93rt_iGJF8r_jV7FYCB1gxdAY7nTUaAnsh8P8HAQ)
5. [PREMIUM FEMALE/FEMALE JUMPER WIRES - 20 X 3" (75MM)](https://www.adafruit.com/products/1951)
6. [PREMIUM FEMALE/FEMALE JUMPER WIRES - 20 X 6" (150MM)](https://www.adafruit.com/product/1950)
7. [PUI Audio, Inc. AS02708CO-WR-R SPEAKER 8OHM 1W 80DB 27X21MM](http://www.digikey.com/product-detail/en/AS02708CO-WR-R/668-1112-ND/1464851)
8. [8x #4 x 0.5” screws](https://www.amazon.com/Screws-Phillips-Stainless-Self-tapping-Quantity/dp/B01LY8W5O4/ref=sr_1_2?ie=UTF8&qid=1515780653&sr=8-2&keywords=%234+x+0.5+self+tapping+countersunk)
9. [Super glue](https://www.amazon.com/Super-Glue-15187-12-Pack/dp/B000LGPD64/ref=sr_1_1_sspa?s=industrial&ie=UTF8&qid=1522249075&sr=1-1-spons&keywords=super+glue&psc=1)

*Water Delivery*

1. [Solenoid](http://www.amazon.com/Vdc-Normally-Closed-Solenoid-Valve/dp/B007D1U64E/ref=pd_rhf_dp_p_img_2?ie=UTF8&refRID=0VP689ZSA23RGAR1QJ6M) valve
2. [Masterflex Tygon E-Food (B-44-4X) tubing, L/S 16, 50 ft.](http://www.masterflex.com/Product/Masterflex_Tygon_E_Food_B_44_4X_tubing_L_S_16_50_ft/HV-06418-16)
3. [Masterflex Tygon E-Lab (E-3603) Pump Tubing, L/S 24, 50 ft.](https://www.coleparmer.com/i/masterflex-tygon-e-lab-e-3603-pump-tubing-l-s-24-50-ft/0650924?pubid=EW)
4. [GVS Easydrop Flow Regulator](https://www.amazon.com/Administration-Easydrop-Rregulator-DEHP-Free-chamber/dp/B00UNZ8N8I)
5. Water [reservoir](http://www.usplastic.com/catalog/item.aspx?gclid=Cj0KEQjwioHIBRCes6nP56Ti1IsBEiQAxxb5Gwes-zsw0myzZCBFYnZjA5MaETTqo2vcvLtHDRPWZ-8aAuCI8P8HAQ&sku=73055)
6. [5 port manifold](https://www.coleparmer.com/i/mn/0646487)
7. [¼” luer lock ring](https://www.amazon.com/Cole-Parmer-Male-luer-lock-nylon/dp/B003NV0ORW/ref=sr_1_3?ie=UTF8&qid=1515783055&sr=8-3&keywords=luer+lock+adapter+1%2F4) male
8. [1/8” luer lock ring male](https://www.amazon.com/Cole-Parmer-Male-luer-lock-Nylon/dp/B003NV2T34/ref=sr_1_2?ie=UTF8&qid=1515783055&sr=8-2&keywords=luer+lock+adapter+1%2F4)
9. [1/8” luer lock ring female](https://www.amazon.com/Cole-Parmer-Female-luer-adapter-Nylon/dp/B003NV2RYA/ref=sr_1_4?ie=UTF8&qid=1515783055&sr=8-4&keywords=luer+lock+adapter+1%2F4)
10. [Water purification tablets](https://www.amazon.com/100-Pack-Purification-Tablets-Aquatabs/dp/B071464NPG/ref=sr_1_4?ie=UTF8&keywords=chlorine%2Bdioxide%2Btablets&qid=1521471647&s=outdoor-recreation&sr=1-4&th=1)

*Sound Attenuating Box*

1. [Box](https://www.go-organize.com/craft-storage-solution/craft-cubes/basic-classics/door-organizer-cube-881.html)
2. 2x [Grommet](https://www.amazon.com/Black-Desk-Grommet-Pack/dp/B000MLCNKW/ref=sr_1_3?ie=UTF8&qid=1516643445&sr=8-3&keywords=1%22+grommet)
3. [Fan](https://www.amazon.com/gp/product/B000LB0M8S/ref=ox_sc_act_title_6?psc=1&smid=ATVPDKIKX0DER)
4. [Female power cord](https://www.amazon.com/HDVDTM-10pack-5-5mm-Pigtail-Female/dp/B00CUKHN0S/ref=pd_sim_421_2?_encoding=UTF8&pd_rd_i=B00CUKHN0S&pd_rd_r=TSQQJV6X0HBPRJFA88VF&pd_rd_w=BDBn4&pd_rd_wg=CH8H7&psc=1&refRID=TSQQJV6X0HBPRJFA88VF)
5. 2” [PVC Slip Flange](https://www.poolpartsonline.com/p-60257-spa-gate-valves-and-slice-valve-flanges.aspx?gclid=CjwKCAjw3_HOBRBaEiwAvLBbovQD3jNwn2b8u27gxH7AeVEt7deEOH7in8wHG1k-fLR5lAwW1JWPUxoC4mwQAvD_BwE)
6. 2” [PVC tube](https://www.amazon.com/gp/product/B002KIF35C/ref=ox_sc_act_title_10?psc=1&smid=A17LJB83C3ZRUZ)
7. [Velcro](https://www.amazon.com/VELCRO-Industrial-Strength-Wide-Black/dp/B00006RSP1/ref=sr_1_1?ie=UTF8&keywords=industrial%20velcro&pebp=1437524309178&perid=01BWNFG7DPDZK50DFNBV&qid=1437524307&s=electronics&sr=1-1)
8. [Wood glue](https://www.amazon.com/Gorilla-6202001-Wood-Glue-oz/dp/B00HDM9I3S/ref=sr_1_2_sspa?s=industrial&rps=1&ie=UTF8&qid=1522248963&sr=1-2-spons&keywords=wood+glue&refinements=p_85%3A2470955011&psc=1)
9. [Super glue](https://www.amazon.com/Gorilla-Super-Glue-Gel-Clear/dp/B00OAAUAX8/ref=sr_1_3?s=industrial&ie=UTF8&qid=1522248992&sr=1-3&keywords=super+glue)
10. [Hygrometer](https://www.amazon.com/dp/B01AEQ9X9I/ref=sxr_pa_click_within_right_4?pd_rd_r=5F15XK03A1DWGHQRS9D1&pd_rd_w=kFpBK&pd_rd_wg=8H8Jz&pf_rd_i=usb%20temperature%20humidity%20sensor%20wireless&pf_rd_m=ATVPDKIKX0DER&pf_rd_p=3008539542&pf_rd_r=QJNZRVS6S4D48JP3PVW0&pf_rd_s=desktop-rhs-carousels&pf_rd_t=301&psc=1)

**Hardware Assembly**

*Central Control Unit*


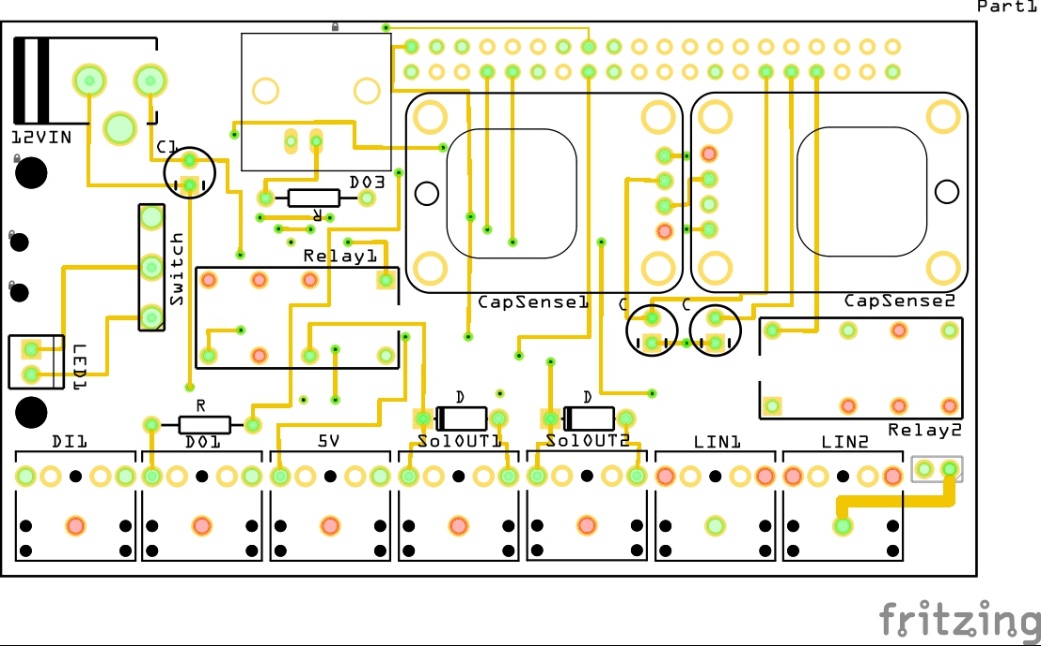


1. Solder the 40-pin header socket (Central Control Unit part #16) to the PCB (Central Control Unit #5) with the pins going upwards and the black plastic connectors beneath the PCB. See the Appendix for a table of Raspberry Pi Pin-outs.


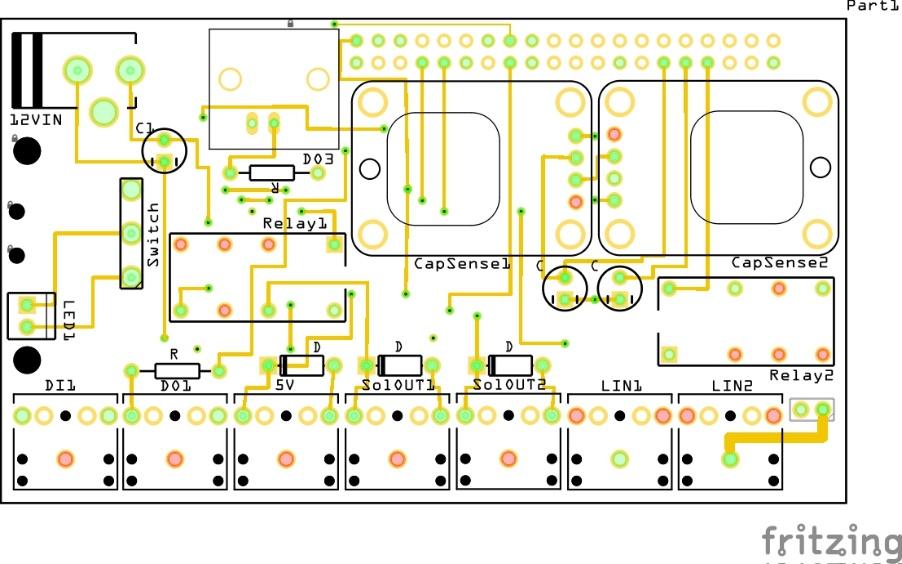


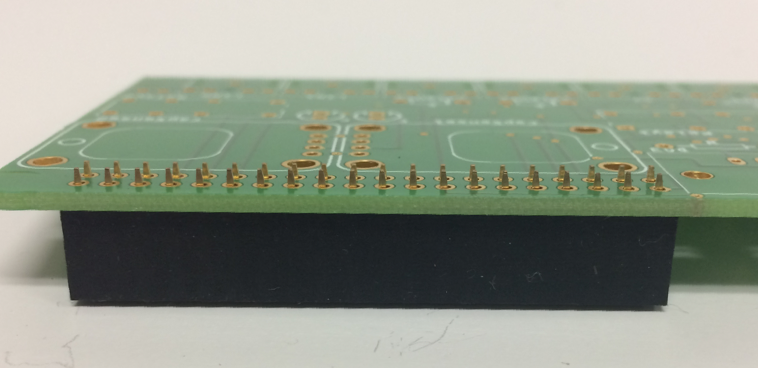


1. Solder the 7 3.5mm stereo jack sockets (Central Control Unit part #7) to the PCB.


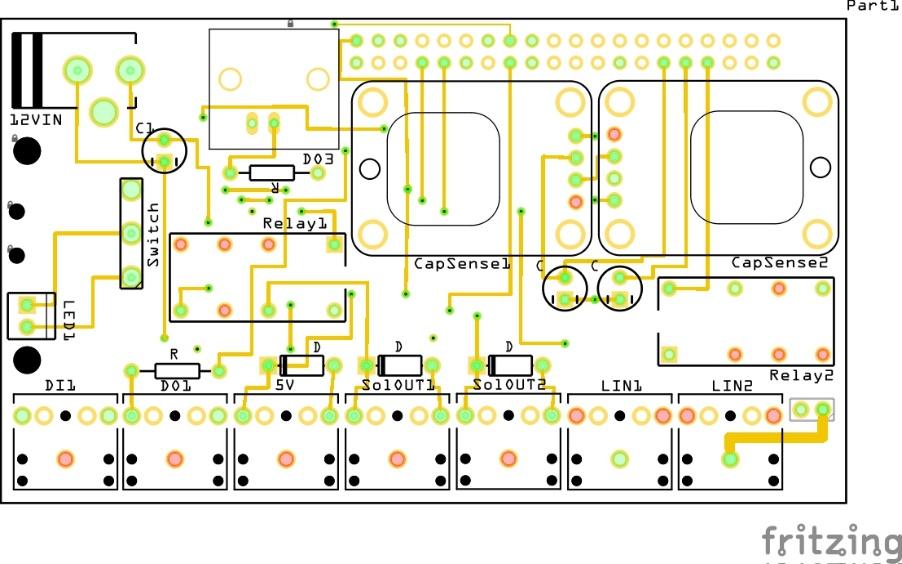


1. Solder the 2 diodes (Central Control Unit part #18) to the PCB. Make sure that the silver stripe on the diode is on the same side as the stripe in the picture (printed in black in the image, printed in white on the PCB).


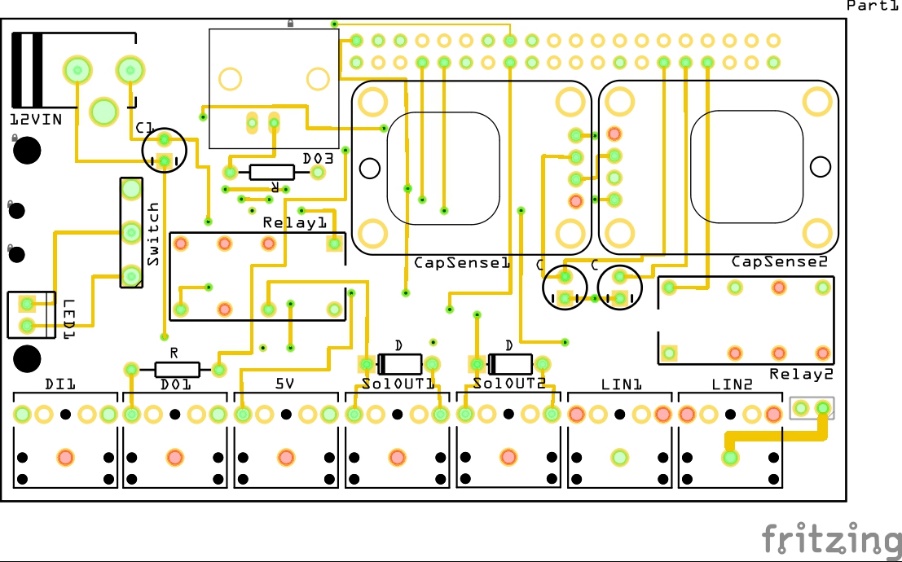


1. Solder 2 resistors (Central Control Unit part #8) to the PCB. If you do not wish to add these to the PCB, strip 2 wires at both ends and solder them into the places that the resistors would have gone.


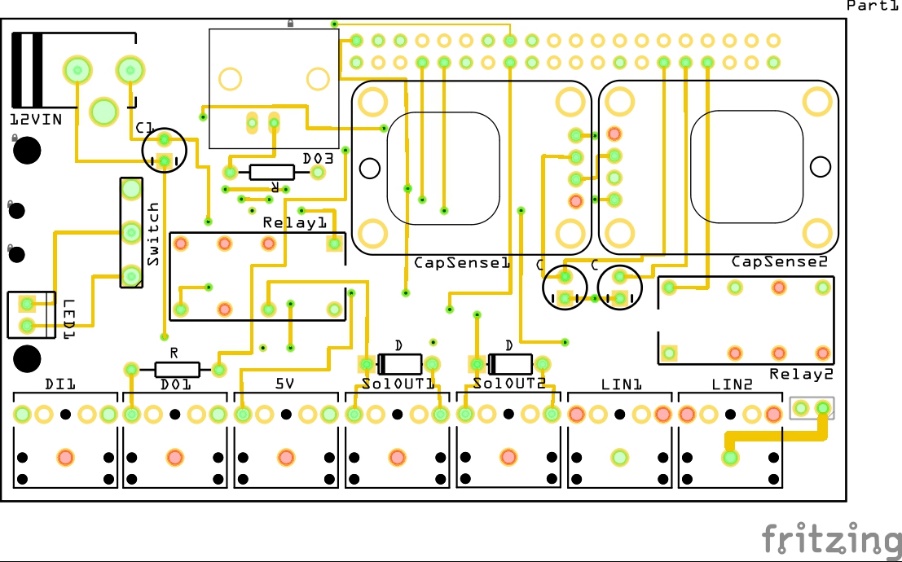


1.
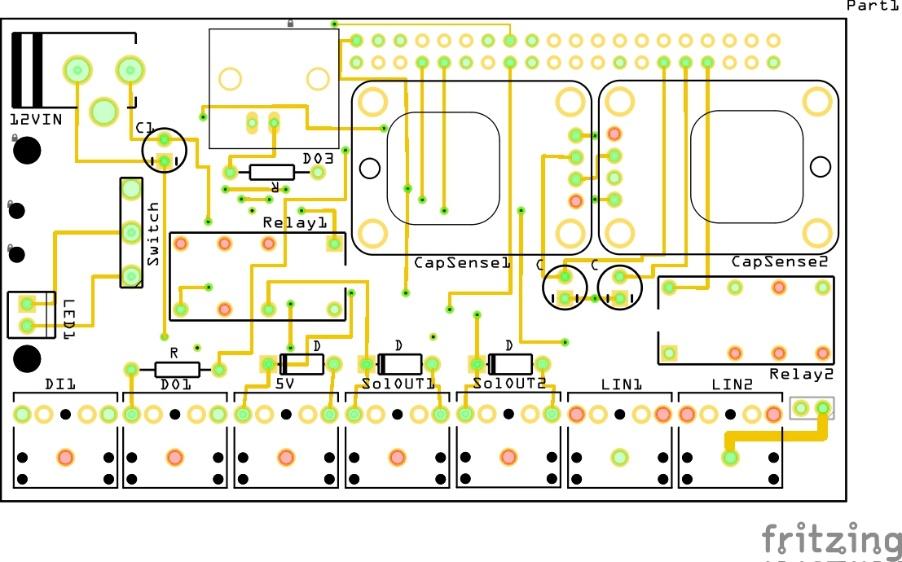
Solder the 3 capacitors (Central Control Unit parts #9 & #10) to the PCB. Make sure that the white stripe on the capacitor lines up with the square shaped connector on the PCB.

4.7uF

1. Solder the DC power jack (Central Control Unit part #6) to the PCB.


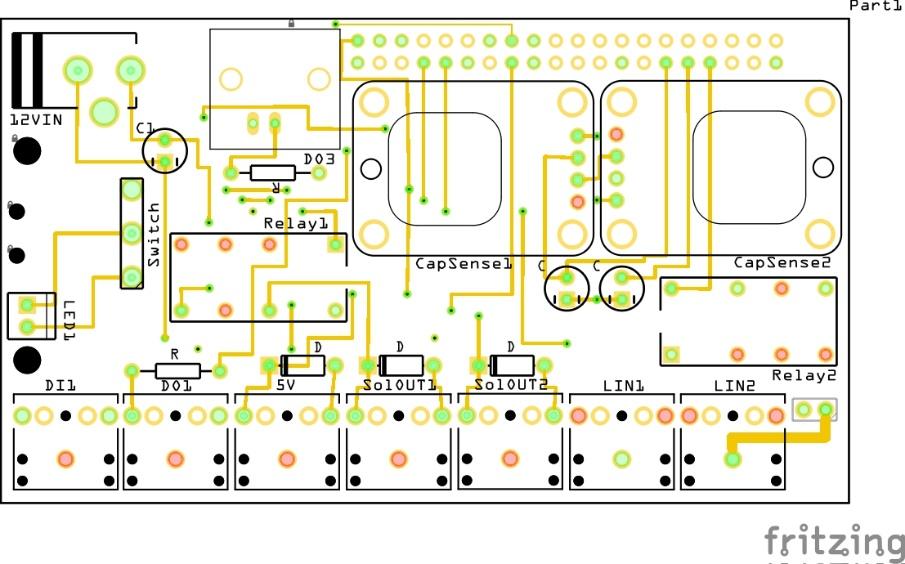


1. Solder the BNC connector (Central Control Unit part #17) to the PCB.


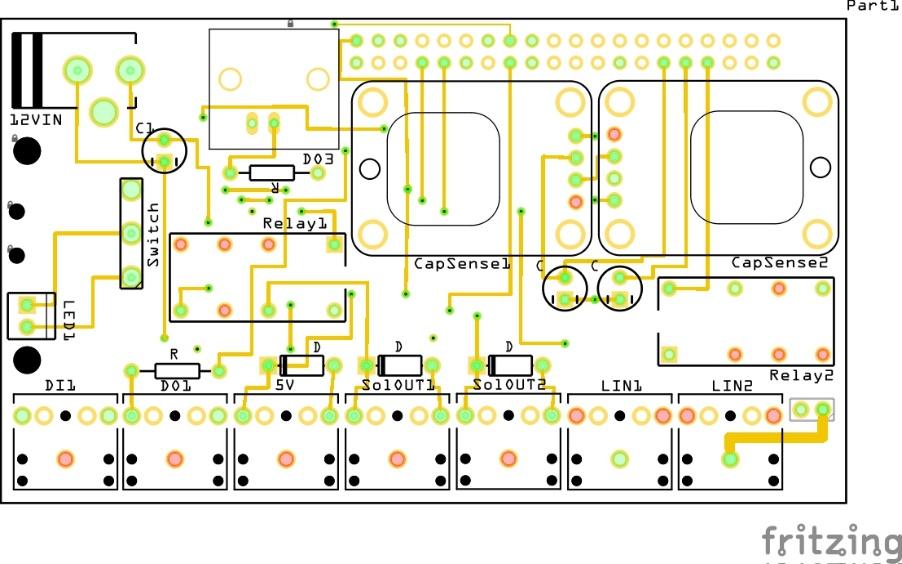


1.
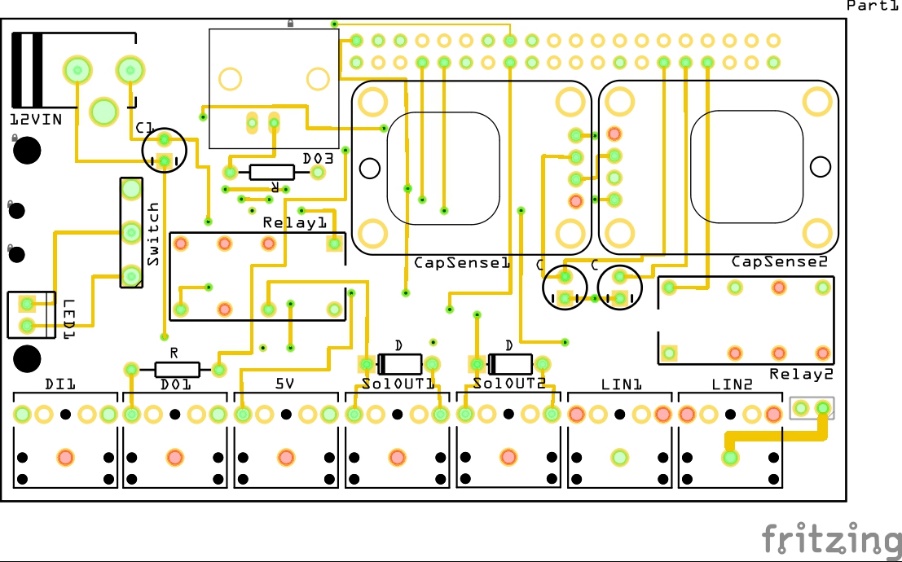
Solder the 2 relays (Central Control Unit part #11) to the PCB.
2. Solder the switch (Central Control Unit part #12) to the PCB.


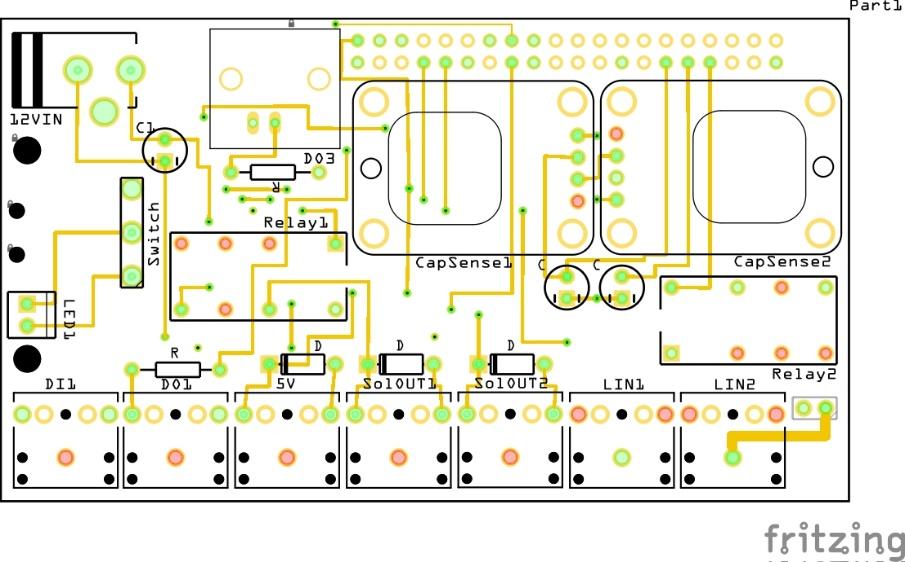


1. Solder the red LED (Central Control Unit part #13) to the PCB.


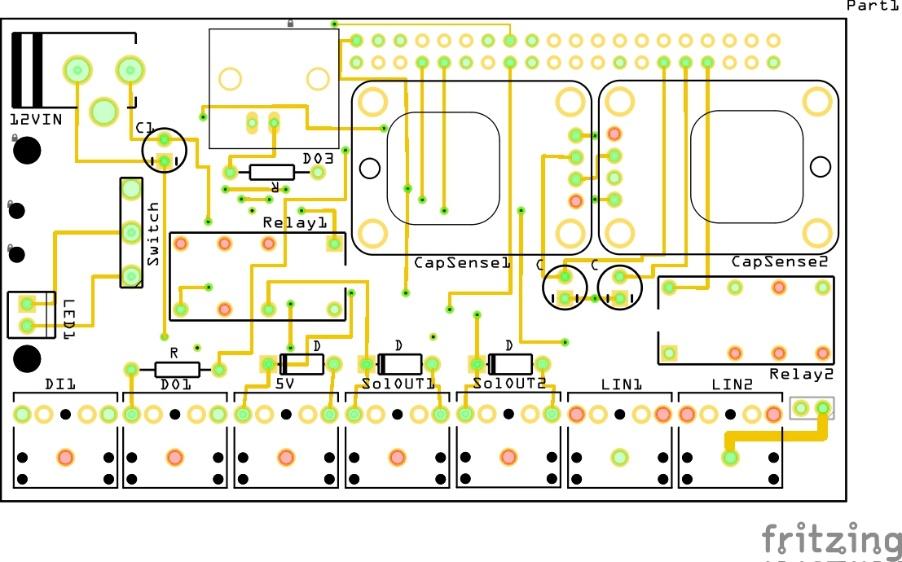


1. Solder 2 connected pins (Central Control Unit part #14) to the PCB next to the relays and the stereo jack sockets.


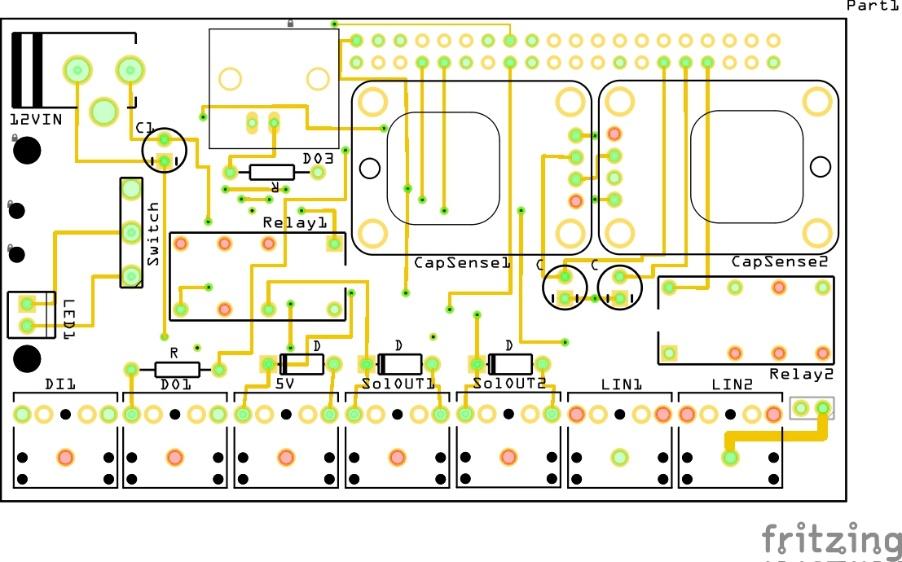


1. Solder 4 connected pins (Central Control Unit part #14) to the capacitive touch sensor (Central Control Unit part #4) such that the plastic connectors are on the same side as the touch pad.


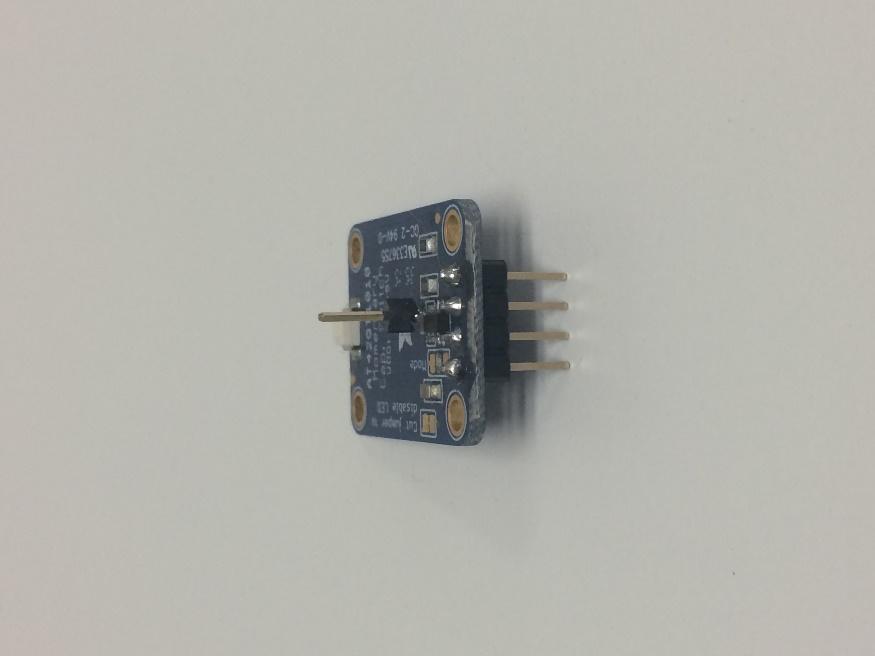


1. Solder a single pin (Central Control Unit part #14) to the middle hole on the capacitive touch sensor with the plastic connector on the opposite side of the touch pad.


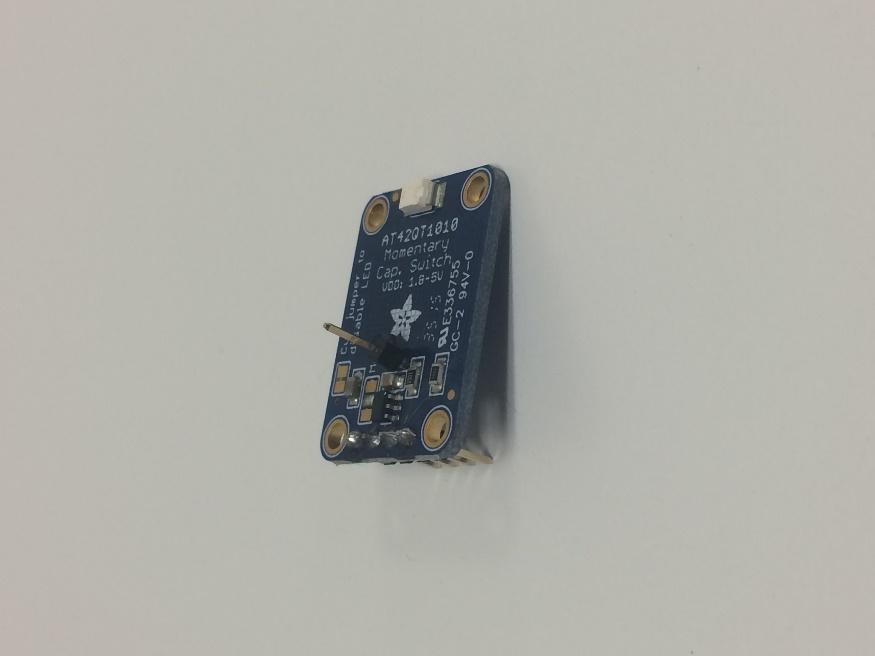


1. Repeat steps 12 and 13 for the second capacitive touch sensor.
2. Solder the capacitive touch sensors to the PCB with the touch pad facing down and the single pin pointing upwards. Make sure that each capacitive touch sensor is soldered such that the sensor is parallel to the PCB with the single pin pointing straight up.


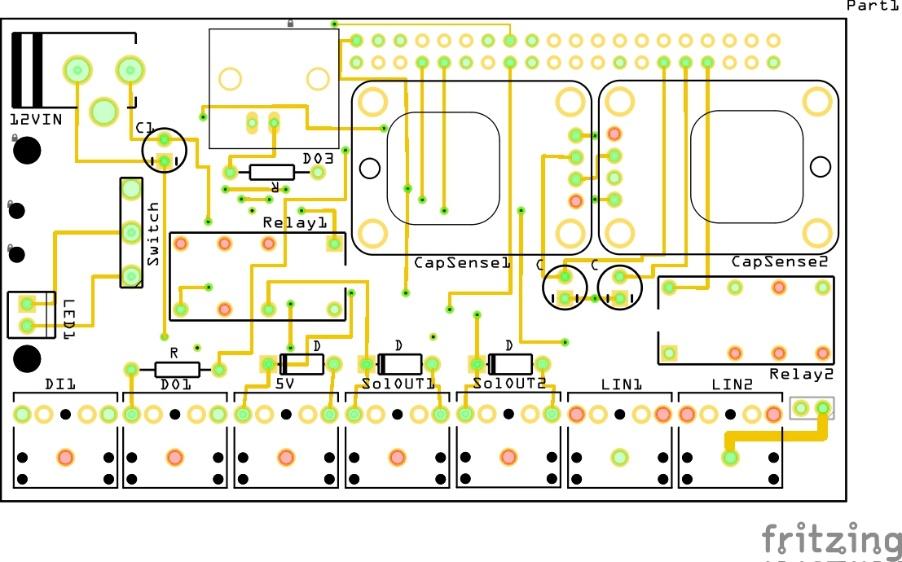


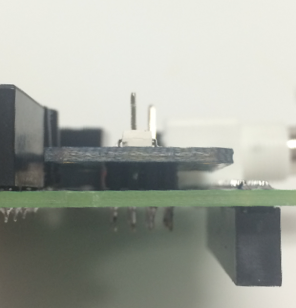

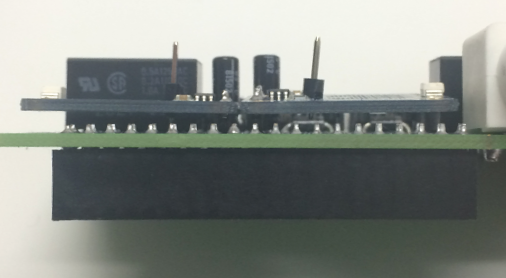


1. Use 2 female jumper wires (Central Control Unit part #15) to connect the 2 pins on the PCB (step 11) to the single pins on the capacitive touch sensors. The left pin should connect to the left sensor and the right pin should connect to the right sensor.


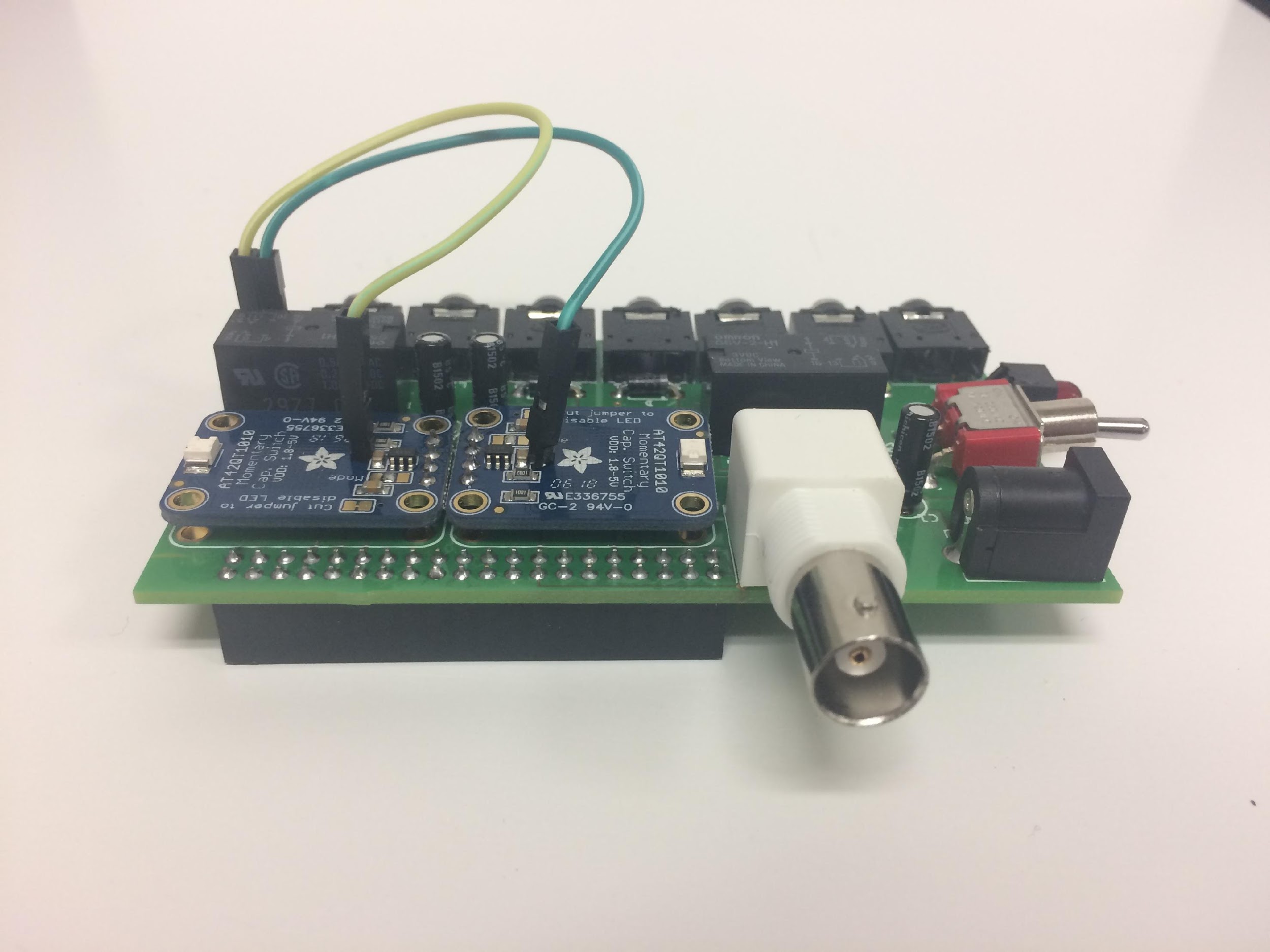


1. Place the now set up Raspberry Pi in the case (Central Control Unit part #1) and screw it in place (only needs 2 screws near the USB ports, the other two are unnecessary). Plug the USB sound card (Central Control Unit part #25) into one of the USB ports.


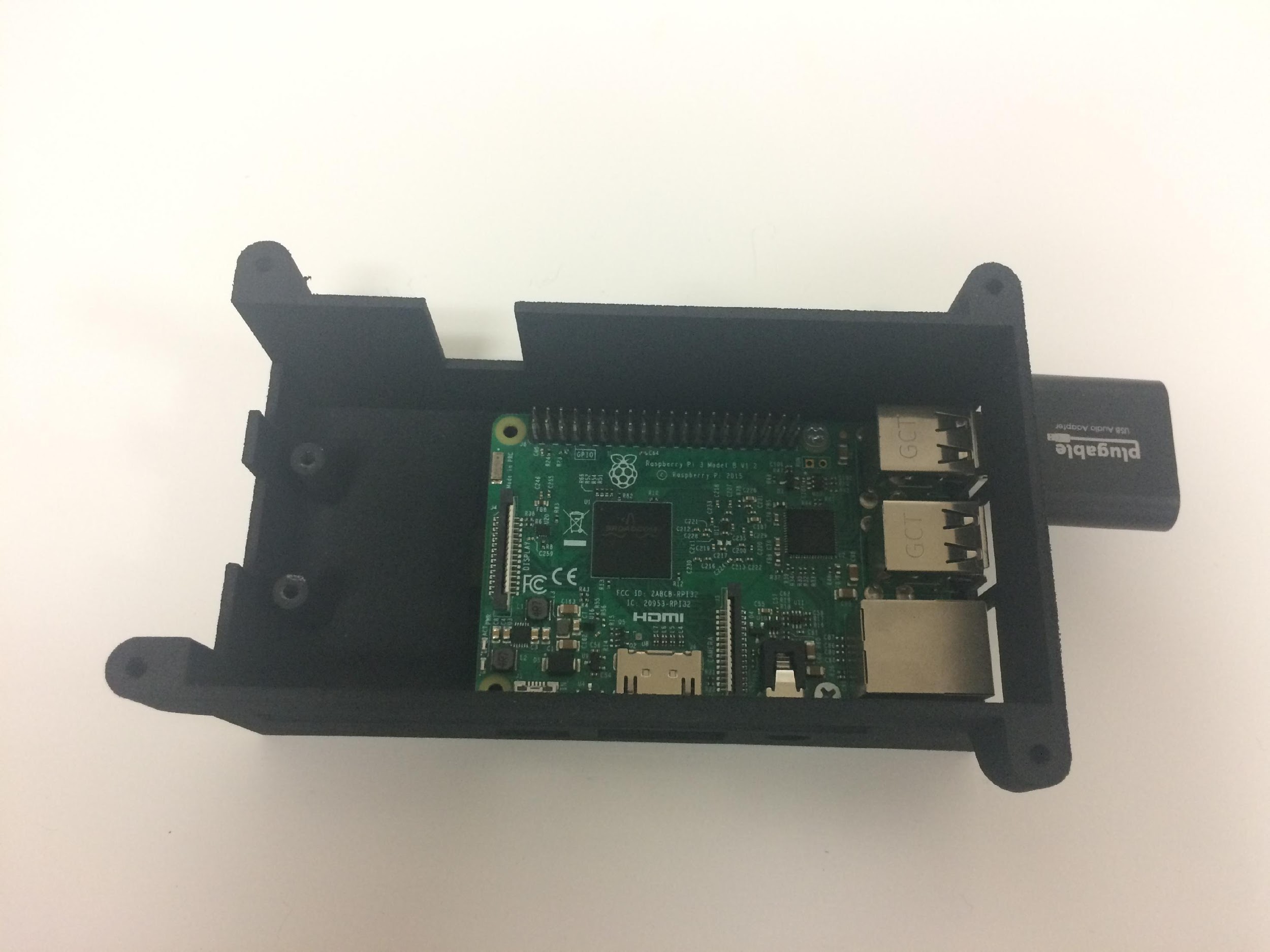


1. Line up the fully assembled CCU PCB with the Raspberry Pi so that the pins on the Raspberry Pi fit into the 40-pin female header socket on the CCU PCB.
2. Screw the CCU PCB to the case with 2 screws near the LED and the switch.


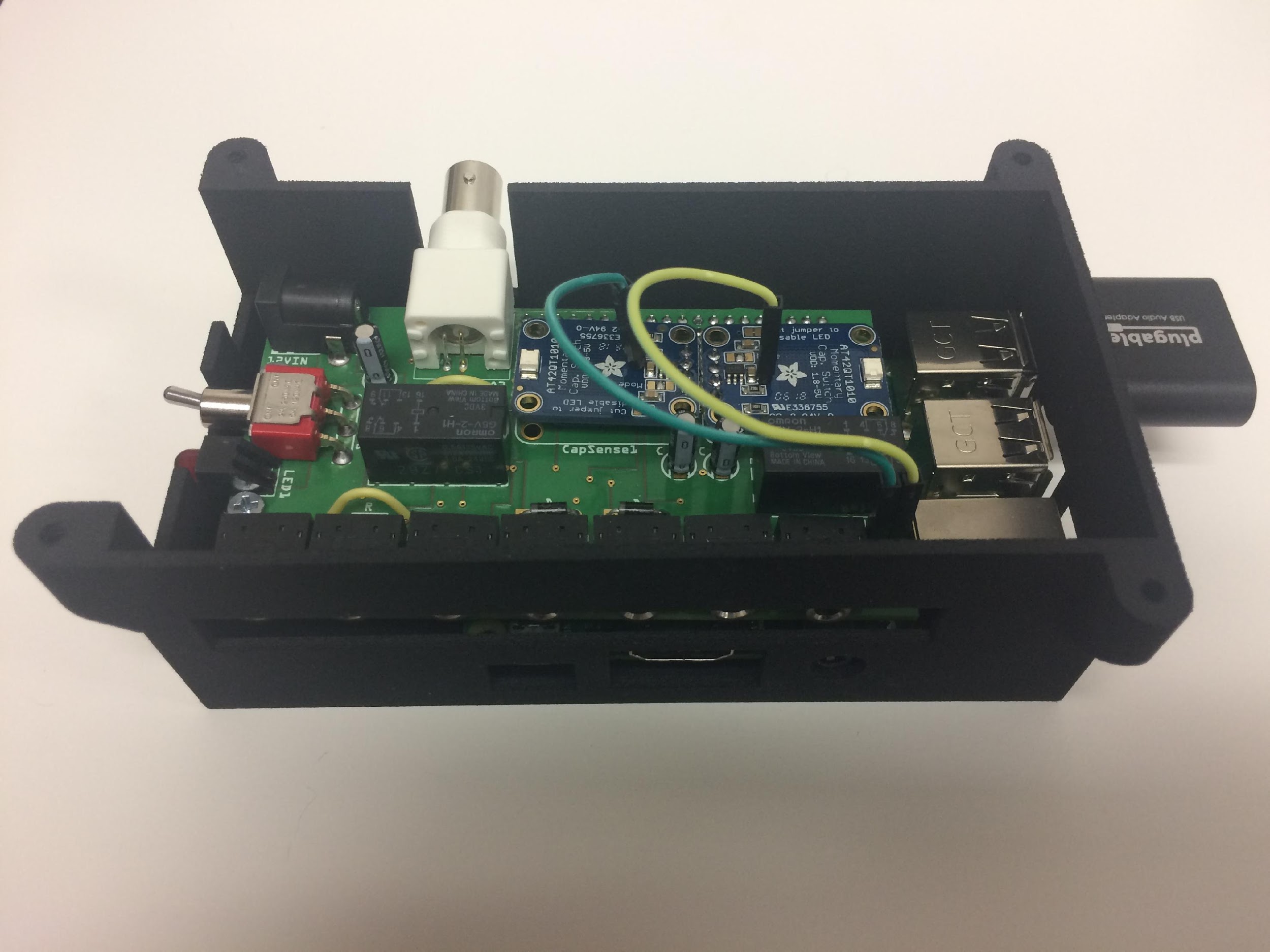


1. Screw on the top of the case.
2. Slide the Behavioral Interface onto the edge of a ‘shoebox’ mouse cage.
3. Connect the Central Control Unit to the Behavioral Interface using audio cables (Central Control Unit parts #21 & #22). One cable should connect each 3.5mm jack on the Behavioral Interface to the Central Control Unit. The 3.5mm jack connected to the speaker goes in the headphones (green) side of the sound card (#22). The 3.5mm jack connected to the power of the amplifier and goes to the 5V socket on the CCU PCB (with the components facing upwards, this socket is third from the left) (#21). The 3.5mm jack connected to the waterspout goes to the socket on the CCU PCB connected to the capacitive touch sensor (with the components facing upwards, this socket is either first or second from the right*) (#22). NOTE: The default uses the first capacitive touch sensor which corresponds to the socket that is second from the right. If the second capacitive touch sensor is used, then the code must be changed to monitor GPIO6 instead of GPIO5.
4. Cut one end of the 12’ audio cable (Central Control Unit part #23) and solder the ends to the leads on the solenoid (Water Delivery part #1).
5. Plug the 3.5mm jack connected to the solenoid to the associated jack on the CCU PCB (with the components facing upwards, the third and fourth jacks from the right are for the solenoid). NOTE: The default uses socket third from the right. If the other socket is used, then the code must be changed to trigger GPIO22 instead of GPIO13.
6. Plug the extension power cord (Central Control Unit part #20) into the Central Control Unit and connect it to the 12V power cord (Central Control Unit part #19).
7. Power on the system by plugging the micro USB cable (Central Control Unit part #24) into the Central Control Unit.
8. See the Appendix for a labeled image of Central Control Unit I/O plugs.

*Behavioral Interface*

1. Solder 5 connected pins to the audio amplifier (Behavioral Interface part #4) with the plastic on top of the amplifier PCB, and solder the screw terminal block (included with amplifier) to the PCB. Make sure the terminals are facing away from the pins like in the picture below.


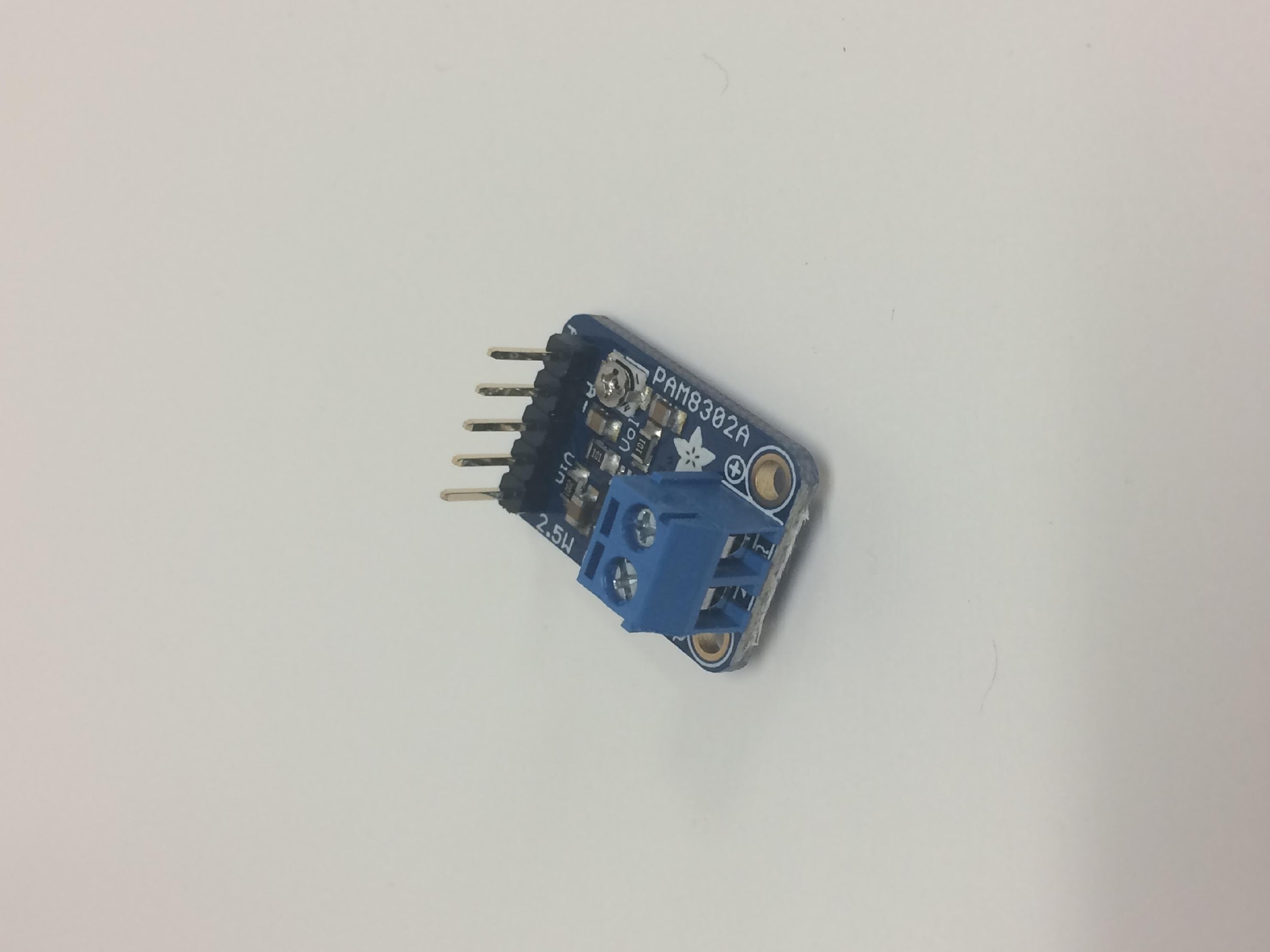


1. Strip one end off of two male jumper wires and solder the stripped end of each wire onto the metal strips on the speaker (Behavioral Interface part #7).


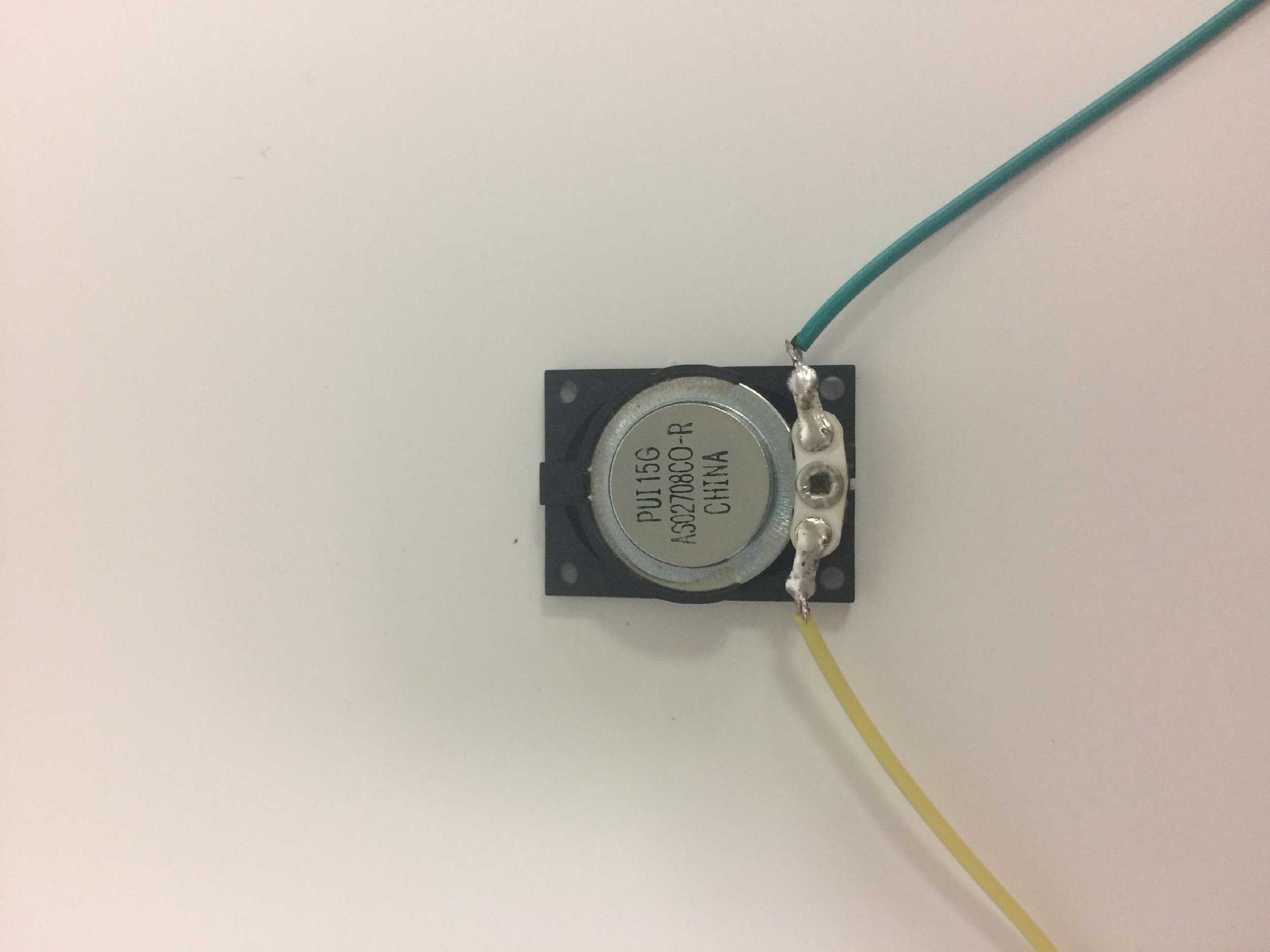


1. Strip one end off four female jumper wires (Behavioral Interface part #5).
2. Solder the stripped ends of the wires to 2 stereo jacks (Behavioral Interface part #3), 2 wires per jack on the left and right leads.


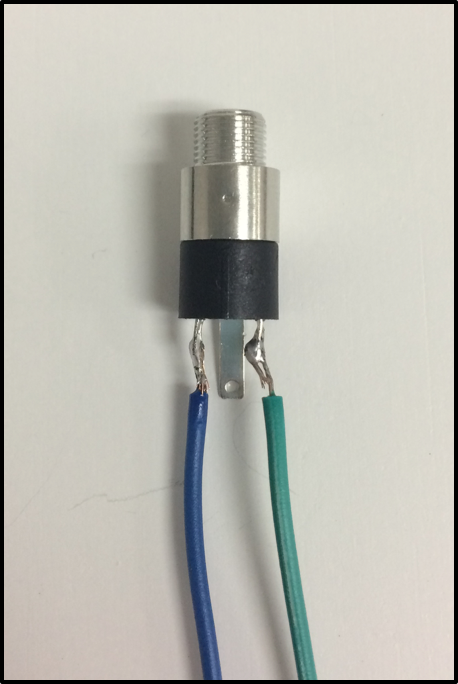


1. Place the amplifier in the rectangular slot closest to the cylindrical portion of the case. For easiest installation, the screw terminal block should be closest to the top of the case. Attach the amplifier to the case by running super glue (Behavioral Interface part #9) around the edge of the amplifier and into the crack between the amplifier and the rectangular slot.


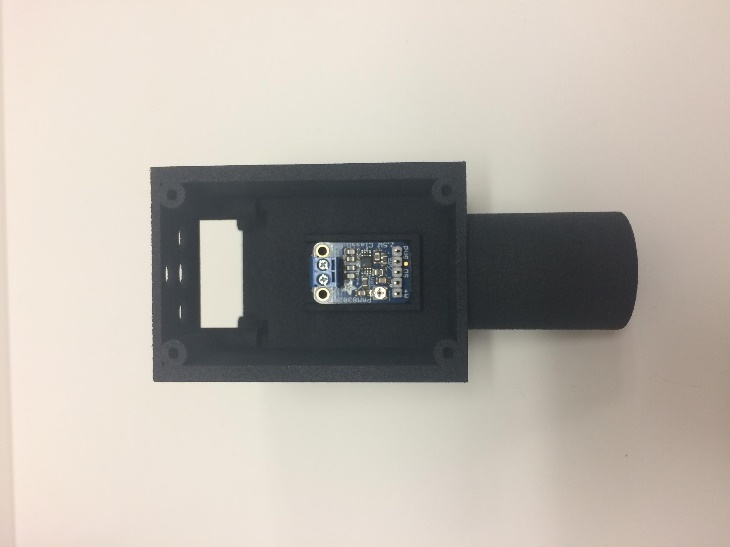


1. Connect the speaker and the amplifier by putting the two wires from the speaker into the screw terminal block on the amplifier.
2. Place the speaker in the remaining rectangular slot in the top section of the case. Attach the speaker to the case by putting super glue in the plastic holes in the corners of the speaker.
3. Strip both ends of a 6” wire (Behavioral Interface part #6) and solder one end to the center lead of a 3.5mm stereo jack (Behavioral Interface part #3).


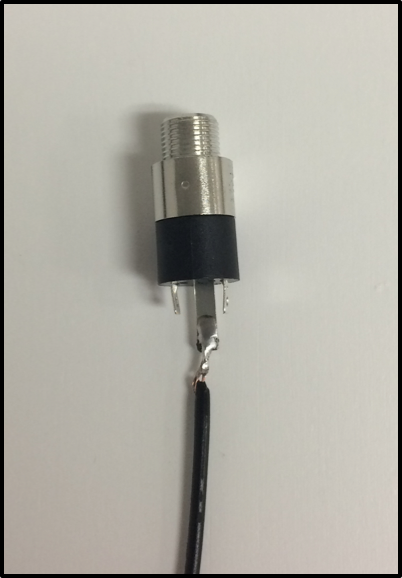


1. Solder the other end of the 6” wire to the peg on the waterspout (Behavioral Interface part #2). NOTE: The solder does not stick very well to the waterspout, so it works best to wrap the stripped portion of the wire around the peg and use lots of solder to hold it in place.


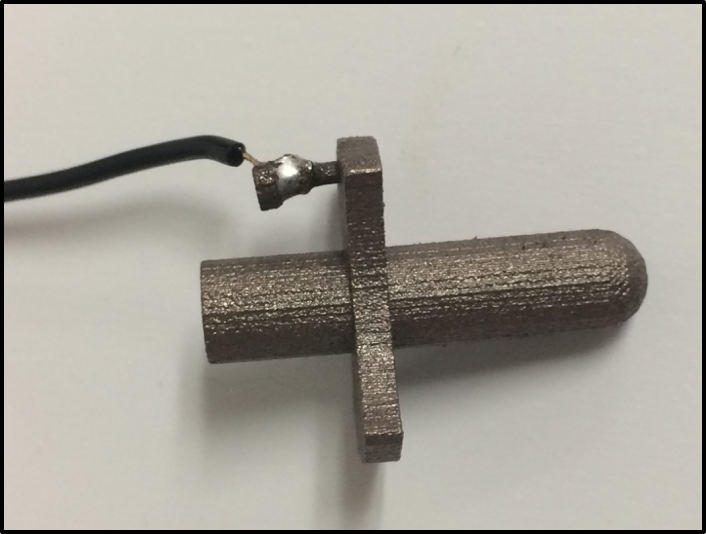


1. Cut a 10” section of tubing (Water Deliver part #2) and stretch the inside of one of the ends to fit onto the waterspout. Slide the tubing onto the end of the waterspout that is on the same side as the peg.
2. Put super glue on the flat surface on the waterspout opposite the peg (indicated below with red arrows) and put the waterspout through the hole at the bottom of the case (Behavioral Interface part #1).


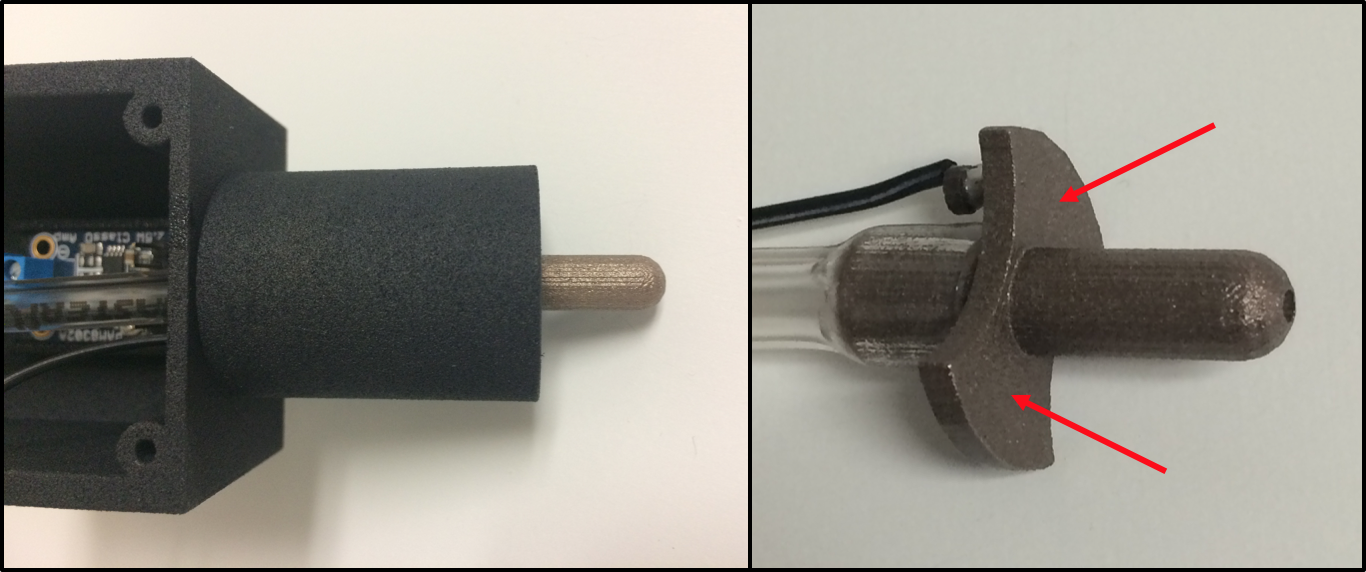


1. Pull the end of the waterspout so the glue gets good contact with the case. Hold until the glue is dry and the waterspout does not move.
2. Run the tubing through the center hole at the top of the case.
3. Place the 3.5mm stereo jack connected to the waterspout through one of the smaller holes and screw it in place using the metal ring (included with Behavior Interface part #3).
4. Place one of the stereo jacks with wires soldered on the left and right leads (from step 4) in one of the remaining holes at the top of the case and screw it in place. Connect the female end of the wires to the VNC and GND pins on the amplifier PCB.
5. Place the last stereo jack in the last case hole and screw it in place. Connect the female end of the wires to the A+ and A- pins on the amplifier PCB. NOTE: The middle pin on the amplifier PCB should not be connected to a wire.


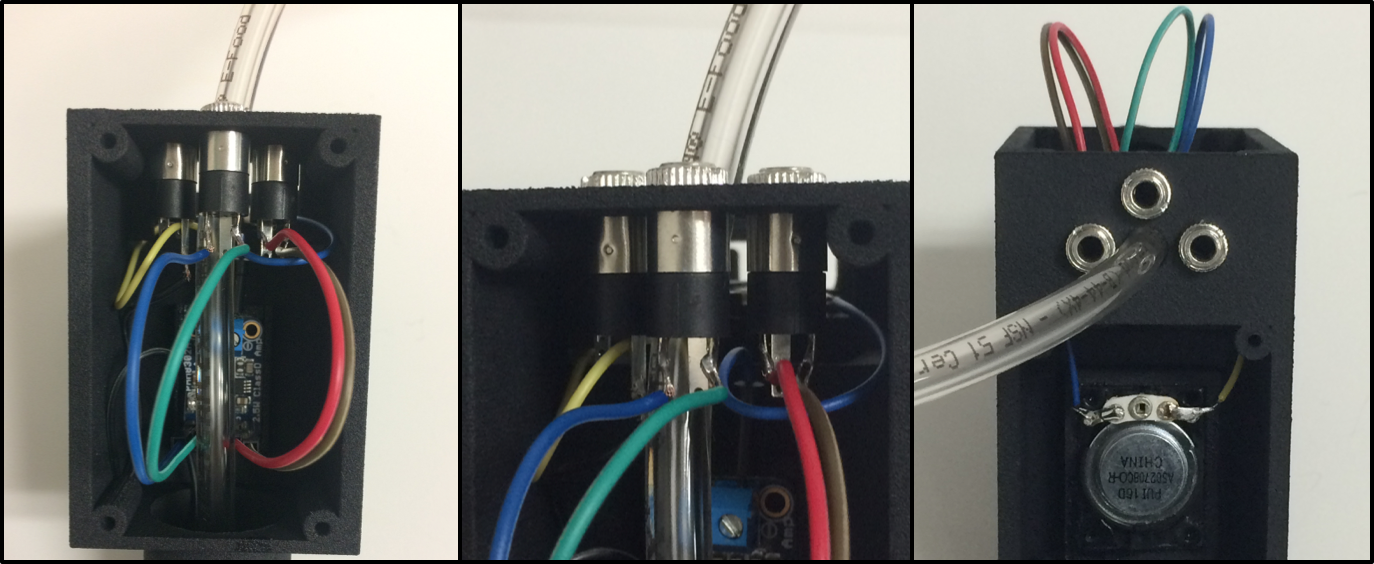


1. Screw (Behavioral Interface part #8) the cover plates onto the case.
2. Attach the water flow regulator (Water Delivery part #4) to the free end of the plastic tube, with the arrow on the regulator pointing toward the case.


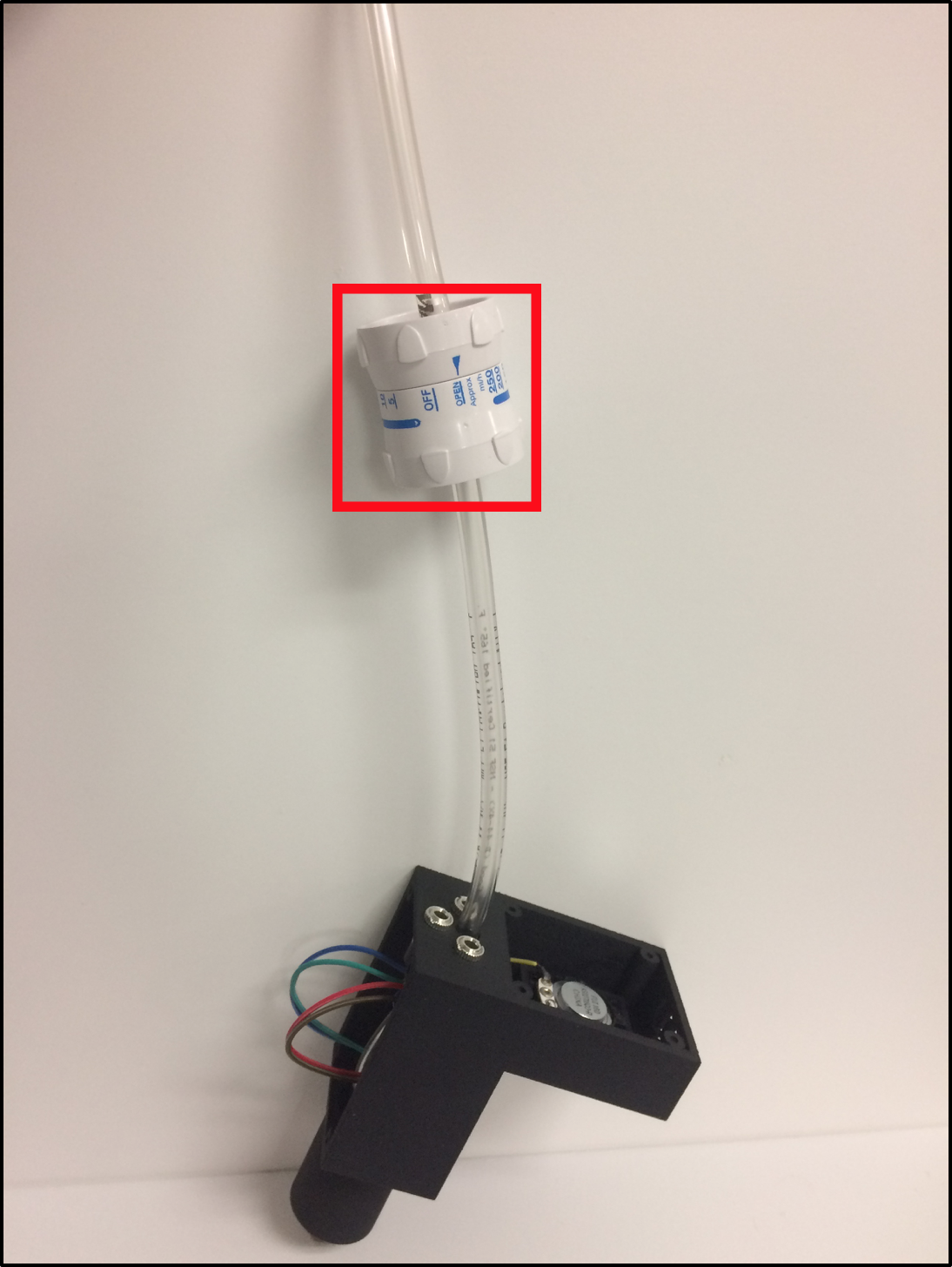


1. Cut a 3” section of tubing (Water Deliver part #2) and attach it to the other side of the flow regulator.
2. Insert and 1/8” female luer lock ring (Water Delivery part #9) into the free end of the tubing.

*Sound Attenuating Box*

1. Assemble acoustic baffle
2. Cut PVC pipes (Sound Attenuating Box part #6) in ~6 inch pieces
3. Line inside of PVC pipe with soft side of Velcro (Sound Attenuating Box part #7)
4. Insert PVC pipe into PVC Slip Flange (Sound Attenuating Box part #5)
5. Trim the excess rim on the bottom of the slip flange using a Dremel to allow it to sit flat


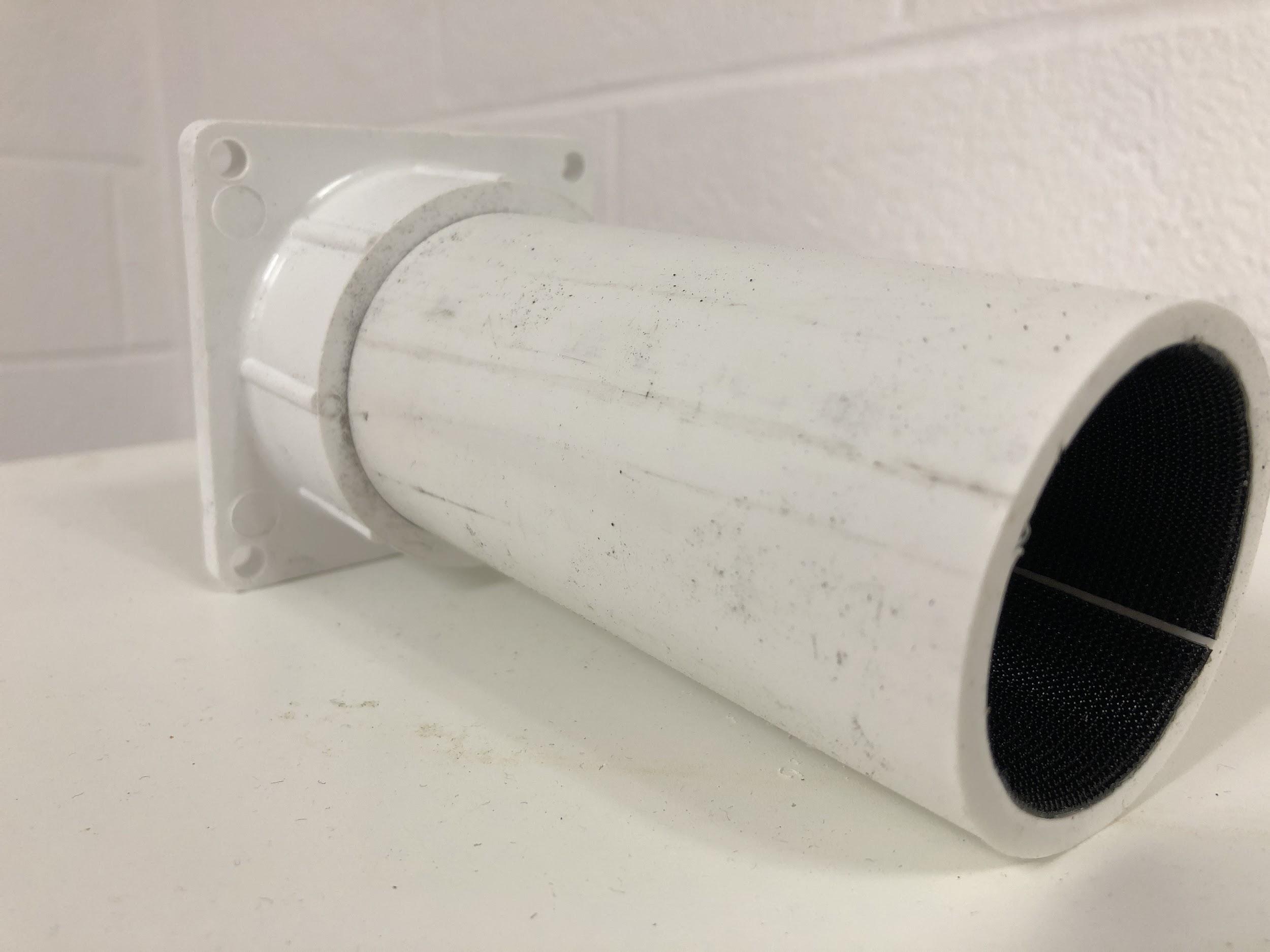

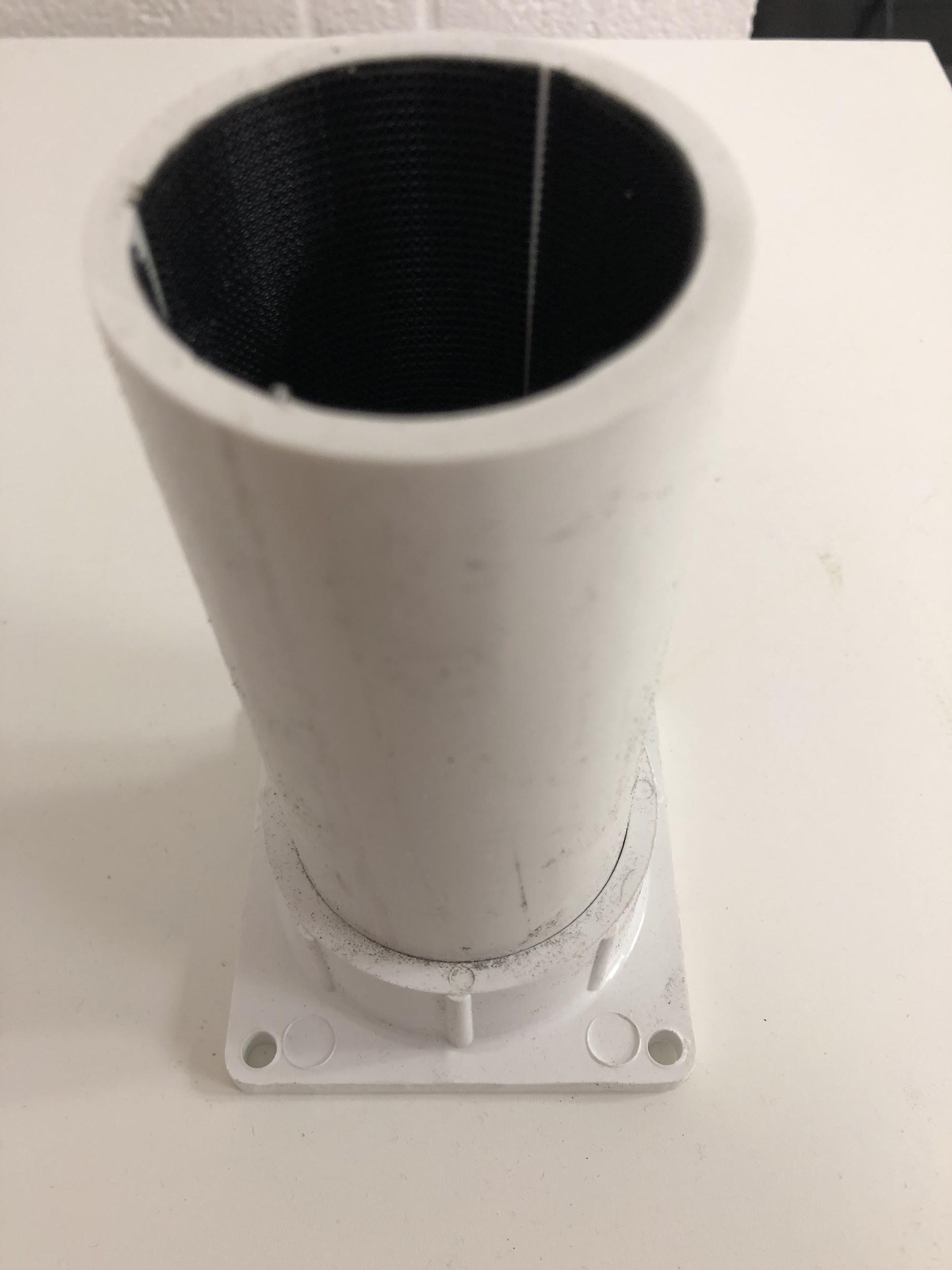


1. Solder together the female power cord (Sound Attenuating Box part #4) and the fan leads (Sound Attenuating Box part #3).

*
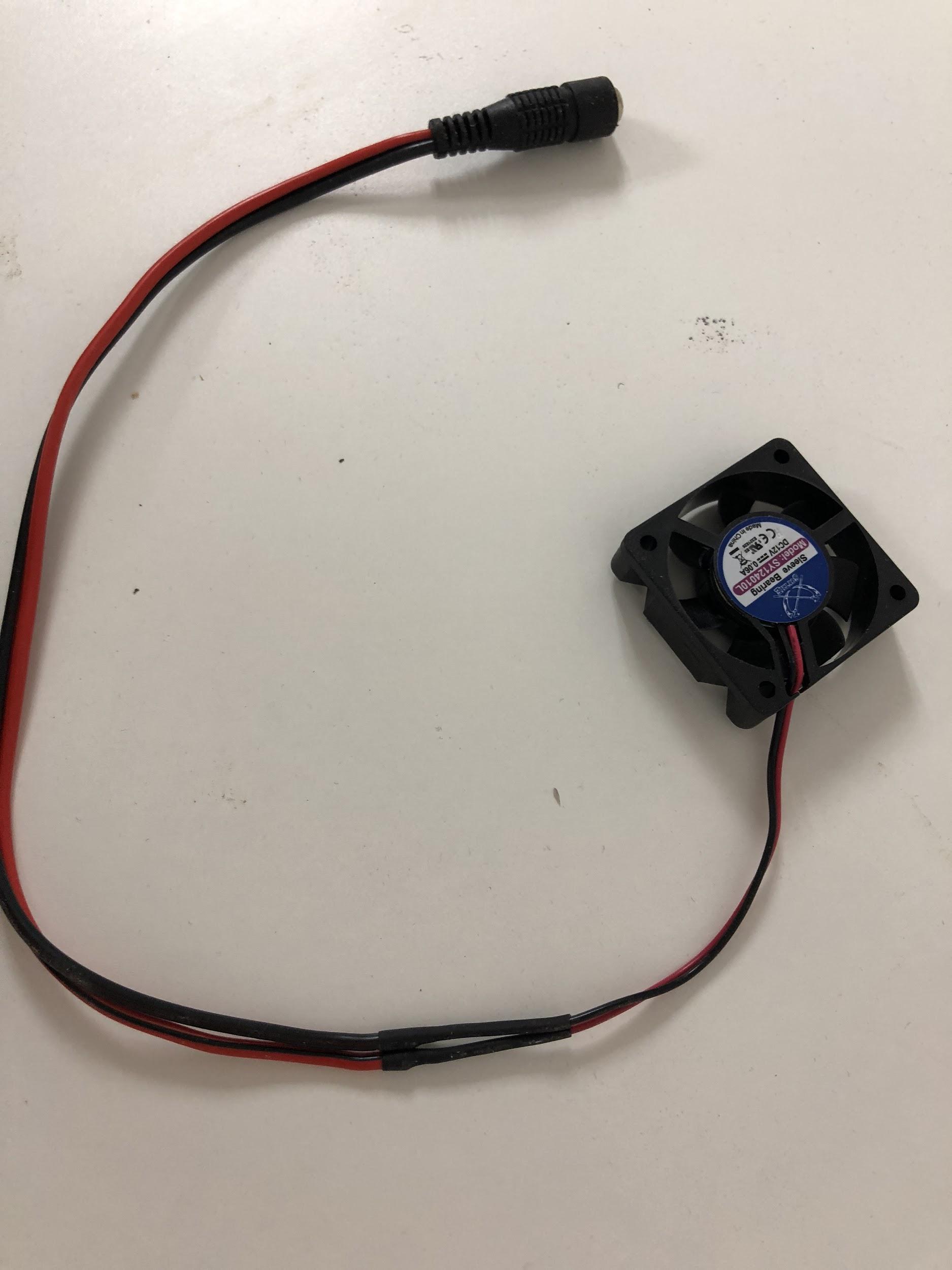
*

1. Make the back-panel of Sound Attenuating Box (Sound Attenuating Box part #1)

1” Grommet

Flange screws

Fan

1. Align slip flange onto upper right-hand corner of the back panel of box; making sure to leave one inch from the top and one inch from the right side of the panel
2. Mark down where the four holes in the PVC flange lie as well as the center point between these points
3. Aligned with center point from step (b), and ~2 inches from the bottom of the panel mark down two more points (one above the other with a 3 inch distance in between)
4. Drill opening at center point large enough to fit circular portion of the [Fan](https://www.amazon.com/gp/product/B000LB0M8S/ref=ox_sc_act_title_6?psc=1&smid=ATVPDKIKX0DER) (Sound Attenuating Box part #3). Make sure that no cardboard from the back-panel touches the fan. The hole rim can be scraped with a razor to clean off loose cardboard.
5. Drill four holes around fan slightly larger than screws to be used to attach PVC flange
6. For the two points at the bottom of panel, drill openings large enough to fit 1” [Grommet](https://www.amazon.com/Black-Desk-Grommet-Pack/dp/B000MLCNKW/ref=sr_1_3?ie=UTF8&qid=1516643445&sr=8-3&keywords=1%22+grommet) (Sound Attenuating Box part #2)
7. Using super glue, glue fan to designated opening (on the side that will be outside of box)
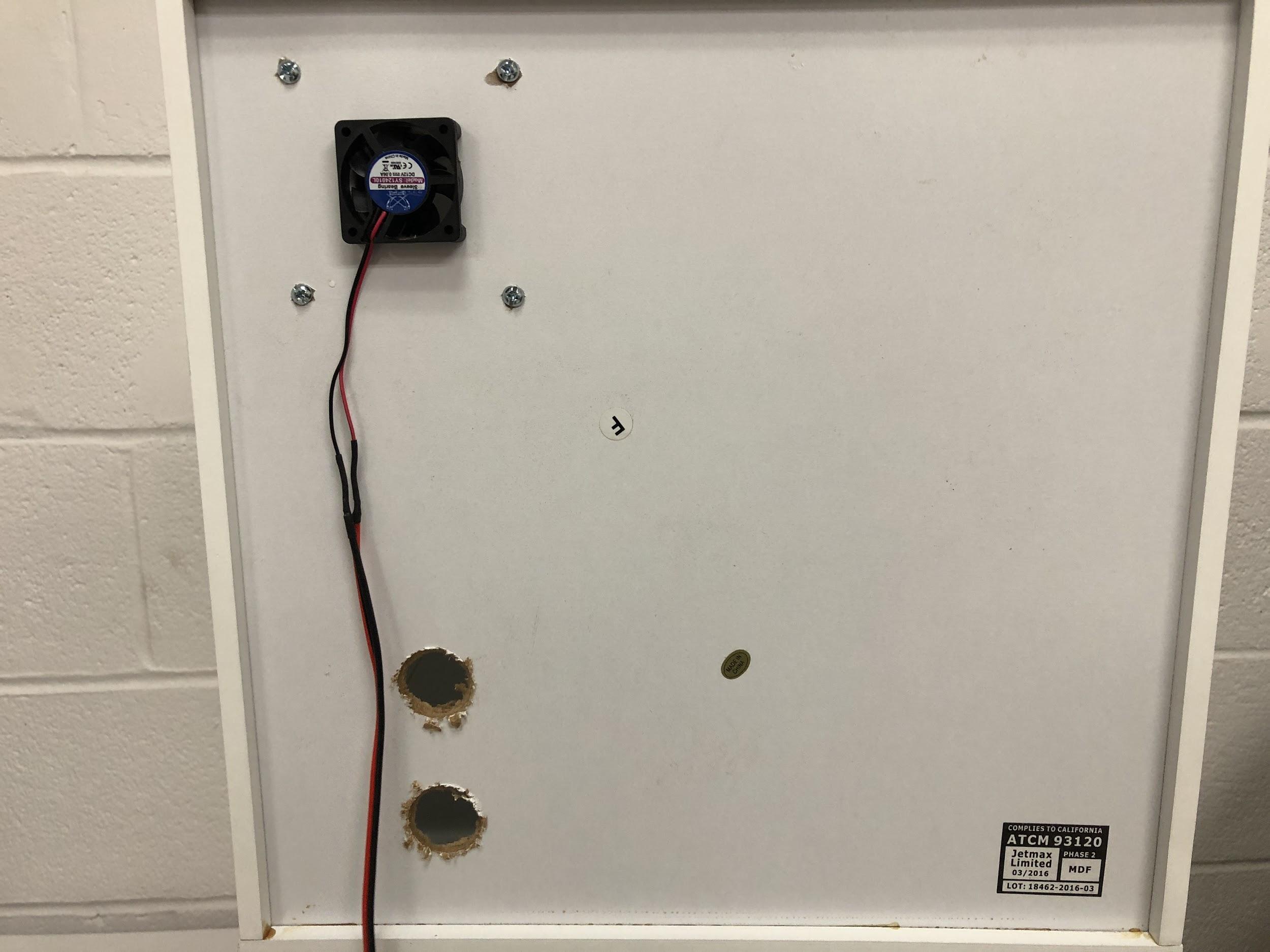

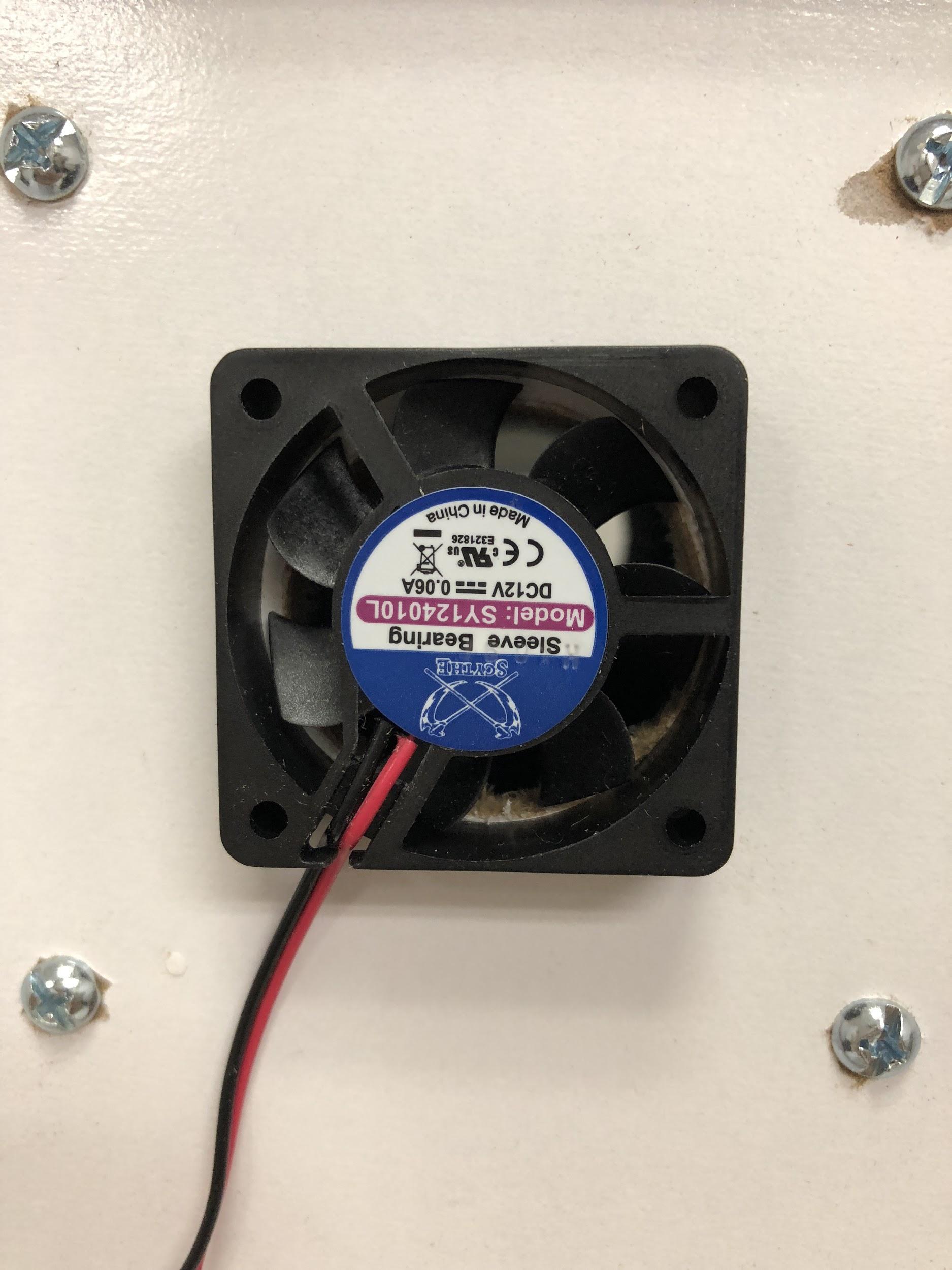

8. Attach PVC pipe/flange using 4 screws and secure with 4 hex nuts


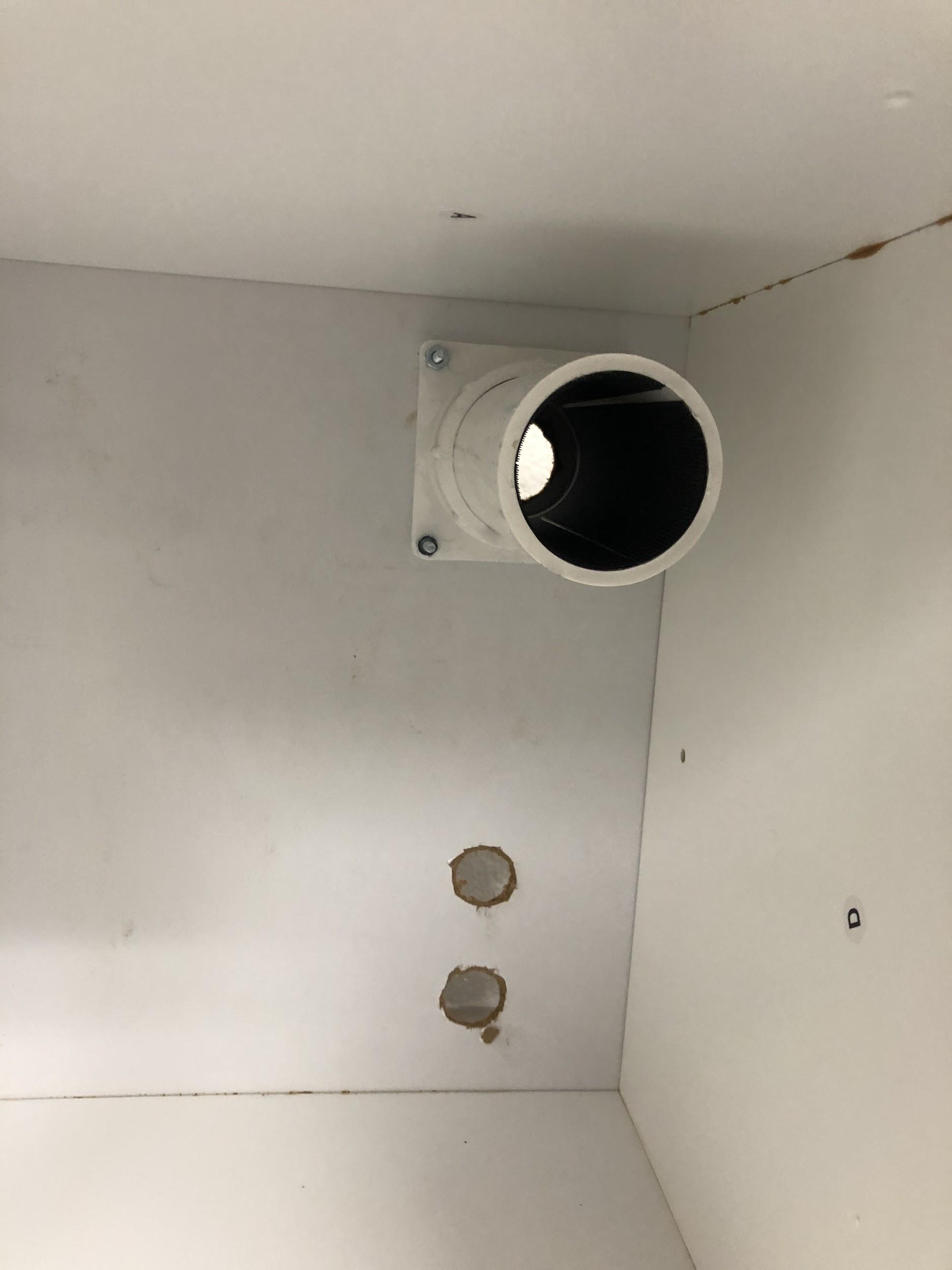


1. Assemble the remaining box according to the instructions included with the box; making sure to place wood glue when attaching panels for added stability

*Water Delivery*

1. Snip the zip ties off the protective tubing on the solenoid.
2. Cut a 6” segment of tubing and one 5’ segment of tubing (Water Delivery part #2).
3. Slide a ⅛” male luer lock ring (Water Delivery part #8) into the 5’ tube and the 6” tubes.
4. Slide the other end of the 5’ tube onto the plastic spout that is perpendicular to the solenoid.
5. Slide the other end of the 6” tube onto the plastic spout that is parallel to the solenoid.
6. Connect the male luer on the 6” tube to one of the ports on the manifold (Water Delivery part #6). Make sure that the port is not off so that water can enter the tube.
7. Cut a 6” segment of tubing (Water Delivery part #2) and slide a ⅛” male luer lock ring (Water Delivery part #8) onto one end and a ⅛” female luer lock ring (Water Delivery part #9) onto the other end.
8. Connect the tube end with the male luer lock ring to the manifold. This will supply water to all ports on the manifold so make sure the valves are situated correctly.
9. Cut a segment of large tubing (Water Delivery part #3) and slide a ¼” male luer lock ring (Water Delivery part #7) onto one end.
10. Slide other end of the large tube onto the spigot of a water reservoir (Water Delivery part #5).
11. Screw the spigot onto the water reservoir. Make sure the spigot is closed.
12. Put 4 water purification tables (Water Delivery part #10) into the jug, fill jug with water, and place it on the top of a shelf. Do not screw top on the jug or water will not flow out.
13. Connect the reservoir to the manifold by twisting the female luer on the tube from the manifold onto the male luer on the tube from the water reservoir.
14. Double check that the valves on the manifold are situated so that the water from the reservoir will not flow out of the manifold and only into ports that are connected to tubes.
15. Open the spigot on the water reservoir. The water should flow through the tubes and stop when it reaches the solenoids.

*ToneBox In Situ (cage top, placement in cage, power, cables)*

1. Slide the Behavioral Interface onto back wall of the cage near the right corner.
2. Using a dremel, cut the bars on a metal cage top so that the Behavioral Interface can slide onto the cage with the cage top on.


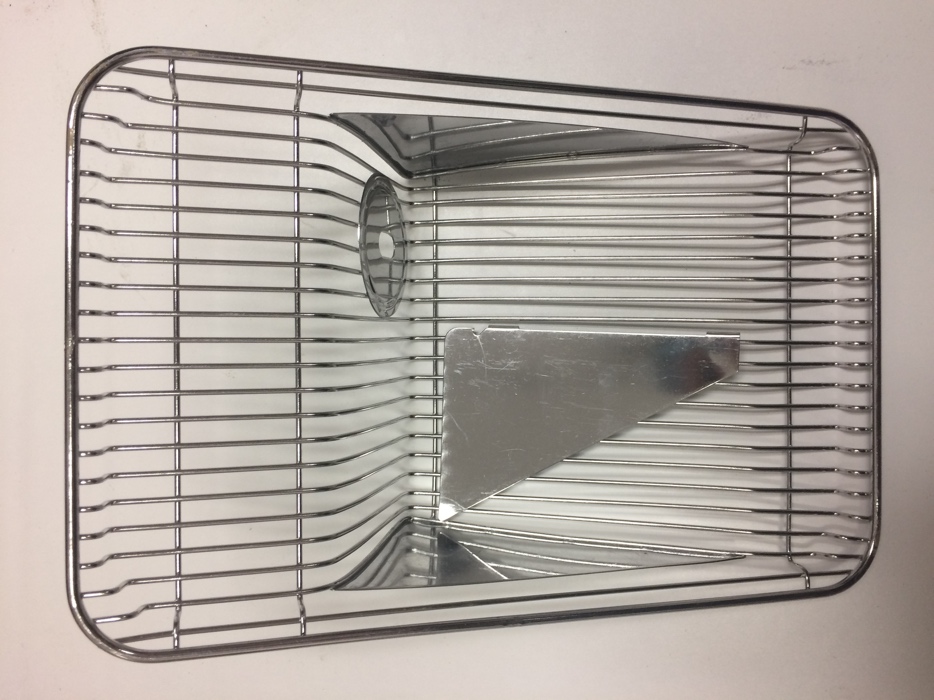

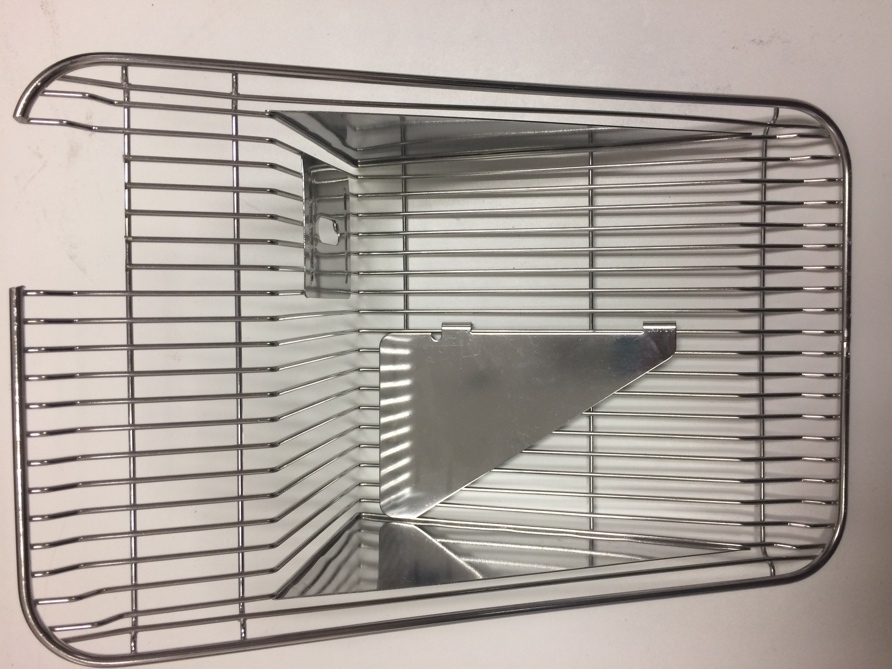


1. Place the central control unit and the cage with cage top and Behavioral Interface into a sound attenuating box.
2. String the long tube from the solenoid, the12’ cable from the solenoid, the 12V DC power cable, and the micro USB power cable through the back of the box to the interior.
3. Connect the solenoid valve to the Behavioral Interface by twisting the female luer from the tube into the Behavioral Interface to the male luer on the tube from the solenoid.
4. Plug the three stereo cables from the Central Control Unit into the Behavioral Interface. The jack connected to the water spout and to the A+/A- pins on the amplifier should get 3’ cables and the jack connected to the VIN/GND pins on the amplifier should get a 1’ cable.
5. Plug the waterspout cable into the Lick Input 1 jack on the Central Control Unit. Plug the amplifier A+/A- cable into the headphone jack on the USB sound card. Plug the amplifier VIN/GND cable into the 5 Volt 3.5mm jack on the Central Control Unit.
6. Plug the solenoid cable into the Solenoid Output 1 3.5mm jack on the Central Control Unit.
7. Plug 12V DC power into the DC power adaptor on the Central Control Unit.
8. Plug a micro USB cable into the Central Control Unit to power on the system. **NOTE: This should be the last step. Do not power on the system until all other cables have been plugged in.**

**Software Installation**

***For the software to run continuously, make sure that the computer does not go to sleep. Turn off any auto-sleep or OS update/restart functions in settings for the computer.***

*Setting up the Raspberry Pi in Matlab*

1. Install the Matlab Support Package for Raspberry Pi. This step only needs to be completed once. If this package has already been installed, search for it by clicking the “Manage Add-Ons” button in the drop-down menu beneath the “Add-Ons” button.
2. Once installed continue with the setup, or if opened to manage, click the gear icon to set up an SD card for the Raspberry Pi. This step will need to be repeated for every new Raspberry Pi.
3. Select the correct type of hardware board, “Raspberry Pi 3 Model B”.
4. When prompted to select an operating system, select “Setup hardware with Mathworks Raspbian image”.
5. When prompted for how to connect Matlab to the Raspberry Pi select the option that says, “connect to LAN or home network”.
6. Insert the SD card (Central Control Unit part #3) into an SD card adaptor and plug it into the computer.
7. Select the option that corresponds to the SD card in the drop-down menu when prompted. Then click “Write SD Card”.
8. After writing the SD card, hit the “Next” button.
9. Remove the SD card from the computer and insert it into the Raspberry Pi. Plug in the USB sound card into the Raspberry Pi as well.
10. Plug an Ethernet cable that is connected to the same network as the computer to the Raspberry Pi.
11. Power on the Raspberry Pi using a micro USB cable. NOTE: Do not power on the Raspberry Pi until steps 9 and 10 have been completed.
12. Hit the “Next” button. Matlab will then find the IP address of the Raspberry Pi.
13. Use that IP address to connect the Raspberry Pi to Matlab. In the command line type the following:

**rpi = raspi(‘IP address’,’username’,’password’)**

The IP address is the number just found by Matlab associated with the device. For a new Raspberry Pi, the username is ‘pi’ and the password is ‘raspberry’.

1. Once Matlab has connected, open up a command line shell for the Raspberry Pi by typing the following:

**openShell(rpi)**

*Raspberry Pi Wi-Fi*

1. In the shell, type the following to update the Raspberry Pi:

**sudo apt-get update**

1. Upgrade the Raspberry Pi:

**sudo apt-get upgrade**

1. Open up WiFi settings:

**sudo nano /etc/wpa_supplicant/wpa_supplicant.conf**

1. This file should only have two lines at the top. Input the following paragraph under those lines:

**network={**

**ssid="WiFi Network Name"**

**scan_ssid=1**

**key_mgmt=WPA-PSK**

**psk="Password"**

**}**

1. Hit Ctrl X, then Y and Enter to save the file.
2. Create new user and password

**sudo adduser ToneBox**

1. When prompted, enter the new password “**thirstymouse**”
2. Ignore the request for new user information by hitting enter and then typing ‘**y**’ when asked if the information is correct.
3. Allow the new user to have root access (make it a sudoer)

**sudo visudo**

1. Change the file to allow the new user “ToneBox” to have access by scrolling down to the line that says “#User privilege specification” and making it look like the following (only the last line is new):

**#User privilege specification**

**root ALL=(ALL:ALL) ALL**

**ToneBox ALL=NOPASSWD: ALL**

1. Hit Ctrl X, then Y and Enter to save the file.
2. Change the hostname of the Raspberry Pi to a name that will be used to identify cages. The hostname must be changed in two files (make sure to type the same name in both files). Type the following:

**sudo nano /etc/hostname**

and change the hostname.

1. Hit Ctrl X, then Y and Enter to save the file.
2. Go to the second file to change the hostname:

**sudo nano /etc/hosts**

and change the hostname which is found in the line that starts with 127.0.1.1

1. Hit Ctrl X, then Y and Enter to save the file.
2. Restart the pi to reset the new hostname

**sudo reboot**

*Raspberry Pi USB Sound*

1. Plug in the USB sound card to the Raspberry Pi. Then list audio devices by typing the following into the shell:

**sudo aplay -l**.

This command should output a list and near the bottom there should be a line that looks like the following:

**card 1: Device [USB Audio Device], device 0: USB Audio [USB Audio]**

1. Change the default audio device to the USB sound card. Type the following:

**sudo nano /usr/share/alsa/alsa.conf**.

Within this file, change the **0** to a **1** in the following lines:

**defaults.ctl.card 0**

**defaults.pcm.card 0**

1. Download the audio player:

**sudo apt-get install mplayer**

1. Restart the Raspberry Pi:

**sudo reboot**

*Raspberry Pi Wi-Fi scanning*

1. When the Raspberry Pi turns back on, there are a few ways to find the new IP address.
   1. Connect a speaker or headphones to the sound card. The Raspberry Pi will say what it’s IP address is while it is rebooting.
   2. Hook the Raspberry Pi up to a monitor via an HDMI cable and plug in a keyboard to one of the USB ports. Log in to the Raspberry Pi after it boots up and then ask for the IP address by typing the following:

**sudo hostname -I**

- 1. Use an IP scanner. NOTE: the matlab program uses an IP scanner to find IP addresses. ***The following instructions are necessary to run the program effectively.***

1. Download an IP scanner from <http://angryip.org/>. Make sure you get the correct one for your computer. Save the application to the same folder that the GUI software is located.
2. Once downloaded, open up the application.
3. Go to the Tools tab and select Fetchers.
4. Select MAC Vendors from the Available Fetchers list and hit the left arrow to transfer the selection to the Selected Fetchers list.
5. Type in the IP range for the WiFi network you wish to scan and hit the Start button. Search for “Raspberry Pi” in the MAC Vendor column to find devices that are connected to the WiFi network. The IP addresses associated with those devices will be listed in the first column.
6. Move application file to the directory that contains the files for the GUI. This allows the GUI to use the application to scan IP addresses.

*Operating the GUI in Matlab*

1. Run ToneBoxGui in matlab.
2. The text box at the top will automatically populate with the current file path that Matlab has open. This is the file that contains the code and figure for the GUI. If this path is where the user would like data to be saved, then press the “Save File Path” button. Otherwise the user can change the file path to where they would prefer to save their data.
3. The “Save File Path” function saves a new directory to that location called “Devices”. If the path that is saved does not already have a “Devices” folder a question box will pop up prompting the user to make sure the correct file path was saved. If “yes” is pressed, then a new “Devices” folder will be created. If “no” is pressed, then the user will be able to change the file path as desired.
4. Press the button that says “IP Scan” to search for the IP addresses of available devices. NOTE: If the IP addresses are already known and you want to shorten the scan time, input numbers in the text boxes to limit the range of the search.
5. In the drop-down menu, select the device that you wish to use. Even if it is showing, you must click on the device for it to be selected. Once a device is selected, the “Water”, “Test Sound”, “Speaker Calibration”, and “Previous Parameters” buttons as well as the “Training Phase” drop down menu become available.
6. The “Speaker Calibration” button is now available. EVERY DEVICE MUST UNDERGO SPEAKER CALIBRATION BEFORE THE FIRST USE OF THE DEVICE. The device does not need to be calibrated before each use, however if a new speaker is paired with a tonebox, then calibration must be done again. The calibration requires a microphone to be hooked up to the output jack of the audio sound card. The microphone should be placed right next to the speaker during calibration and calibration should occur in the same environment that a mouse would train in. Make sure to not make any noise during this process. The calibration will produce and filter eight tones that will be saved onto the Raspberry Pi for use during the training tasks. NOTE: This button will become unavailable once the “Start” button is pressed.
7. The “Water” button is now available. You can press this button to see that the water will run unobstructed and at the correct rate when needed. To adjust the rate, turn the flow regulator while watching the water drip from the spout until the water drips out at the desired rate. Press the “Water” button again to turn off water flow. NOTE: This button will become unavailable once the “Start” button is pressed.
8. The “Test Sound” button is now available. You can press this button to test that the speaker is functioning correctly. The sound will be a 1 kHz test tone played for one second followed by a one second pause and repeating until the “Test Sound” button is pressed again. NOTE: This button will become unavailable once the “Start” button is pressed.
9. The drop-down menu that says “Training Phase:” is now available. Select the phase you wish to run on the selected device. The options are “Habituation”, “Shaping”, “Detection”, and “Discrimination”. NOTE: This menu will become unavailable once the “Start” button is pressed.
10. Once a phase has been selected, the boxes that offer choices for tones will become available. NOTE: Habituation does not play sound so no tones will become available. Additionally, the “Nontarget Tones” will only become available if “Discrimination” has been selected and the “Silent Trials” radio button is only available for “Detection” and “Discrimination”.
11. Select the tones and tone levels you would like to play during the training task. Select “Silent Trials” if you would like to include them in the training task. NOTE: Not all options will be available for each phase.
12. Type in the Start Time and Stop Time for when you would like the training to occur, for example if you would like to restrict training to the active hours of a mouse’s circadian rhythm. If you would like training to occur continuously, then type in the same number for Start Time and Stop Time.
13. Type in how many minutes long you would like each block and inter-block interval to be in Block Time and IBI Time.
14. Press the “Set Params” button. NOTE: If you decide to change the parameters for the selected device, you must hit the “Set Params” button again otherwise only the previous settings will be used.
15. If you would like to use the parameters that you selected for the last time that device that was started, press the “Previous Params” button and it will populate the previous settings. Hit the “Set Params” button to lock in the settings. NOTE: This button will become unavailable once the “Start” button is pressed.
16. The “Start” button is now available. Once this button is pressed, the training task will start on the selected device. The task options on the GUI will reset so that you can start another device if desired. The device that is running will now say “In Use” next to the name and IP address in the “Raspberry Pi IP” drop down menu. The “Stop” and “Pause All” buttons are now available.
17. The tasks are programmed to record a ‘performance.mat’ file in a folder with the same name as the device. Once the first instance of this file is saved you will be able to start additional devices (repeat steps 5 – 16, skipping step 5 if the speaker is already calibrated, to start more devices). DO NOT CLICK ON A DEVICE THAT SAYS IT IS “In Use”.
18. To stop a current device, click the tab that is displaying the device’s data. Then hit the “Stop” button. The program will run one more trial after the “Stop” button is pressed to ensure no data is lost, unless the program is in between trials or in between blocks in which case the program will be stopped immediately. The tab with the device’s name will disappear once the it has finished running. NOTE: Due to the no-lick period before a trial, it will take anywhere between 5 and 30 seconds for the device to stop if the mice are actively engaging.
19. If a device takes longer than 60 seconds to stop once the “Stop” button has been pressed, a dialogue box will pop up. The dialogue box will ask if the device is still running or if it has failed. If the mice are licking the water spout a lot and preventing the next trial from running, then click the “Device is still running” option. If the device has failed, select the “Device has failed” option.
20. If the “Device has failed” option was selected, a “Terminate” button will appear under the “Stop” button. Click on this button to stop the failed device. MAKE SURE YOU ARE STILL ON THE CORRECT TAB! Another dialogue button will pop up asking to reconfirm the termination. Select “Yes” and the device’s tab will disappear. NOTE: The data from this device will be saved as performance.mat until the next time the device is run and it will be resaved with the time-stamp and a message that says STOP_ERROR. The “Terminate” button will then disappear.
21. Repeat steps 18-20 to stop all the devices.
22. Pausing all the devices is a necessary feature when cages must be cleaned/changed and the water delivery system must be refilled. Press the “Pause All” button and all the devices that are currently running will be paused. While paused, no data is being recorded and no tones are being played so the BI can be moved/touched without skewing the data. NOTE: The “Pause All” button will disappear and the “Resume All” button will take its place.
23. Press the “Resume All” button to resume the experiments. NOTE: The “Resume All” button will disappear and the “Pause All” button will appear again.
24. Data is saved within the “Devices” folder that is located in the file path that was selected in the first step. Within the “Devices” folder there are folders for each of the devices that were run or have been run in the past. The specific folders for each device contain the performance data files which have been time stamped at the end of the last trial after the stop button is pressed.
25. To view the data collected by each device, run dataGraph in matlab.
26. Click the “Check Devices” button to check the status of the currently running devices. The buttons on the left will populate with the names of the running devices and they will be assigned a color based on their activity. A legend will appear at the bottom for color interpretation.
27. Click on the button for one of the devices and the text box at the top will populate with that device name. The current data will also be graphed. The drop-down menu will populate with the data files associated with that device (listed from newest to oldest). NOTE: If the device is currently running, the top file will be “performance.mat”. Also, the data is not plotted in real-time. You must click the device button again or the “Graph Data” button in order to update the data being displayed.
28. If you would like to graph data from a device that is not running, simply type in the name of the device in the text box and hit the Enter key. The drop-down menu will populate with the data files corresponding to that device (listed from newest to oldest). Select the file you wish to graph and then click the “Graph Data” button.
29. Click the “Error Notification” button in order to receive an email notifying you when a device has failed. Input the necessary information when prompted. NOTE: The “Error Notification” button will turn black when it is activated. You must deactivate the “Error Notification” button in order to interact with the GUI again. The button will turn white/gray when it is deactivated.

Appendix

*Raspberry Pi pin-outs*

*
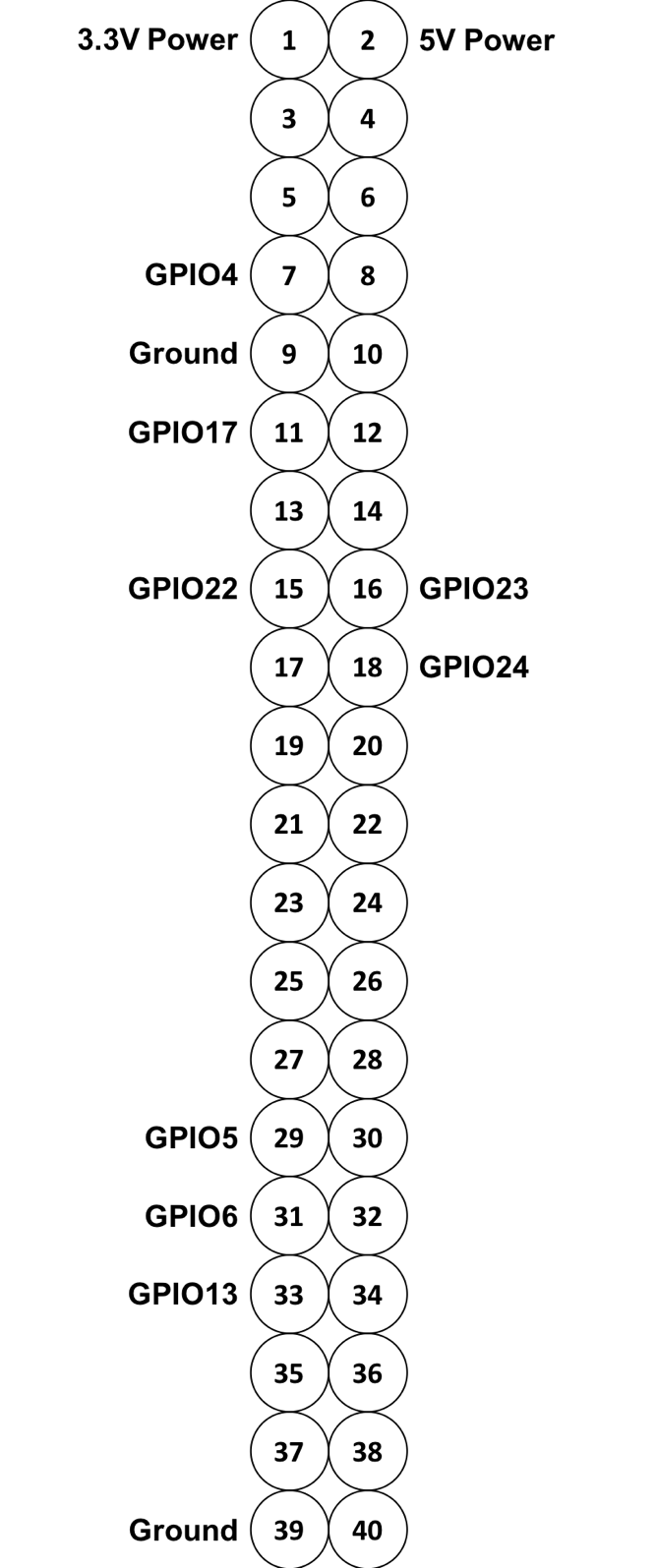
*

*Central Control Unit I/O*


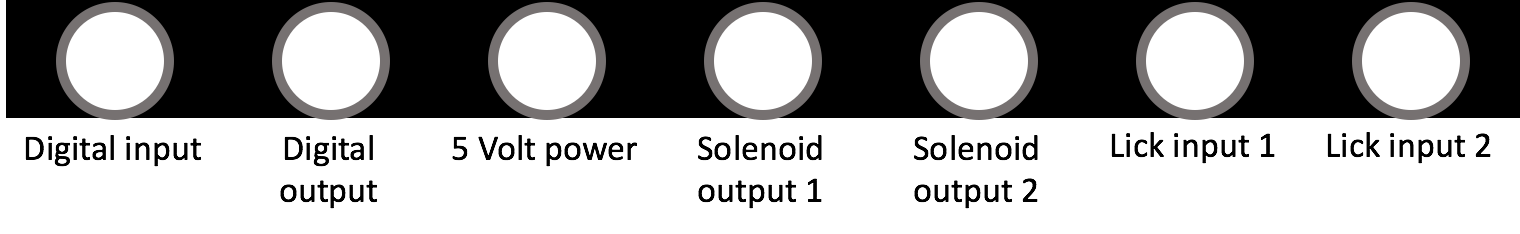


*Training Phase Parameters*

Habituation

Trial length = 10 seconds

Conditioning water probability = 100%

Conditioning water onset time = 0 seconds

Conditioning water duration = 5 seconds

Inter-trial interval range = 30-300 seconds

Training block duration (should always be 1 hour) = 1 hour

Inter-block interval (should always be 1 hour) = 1 hour

Response window = 0-10 seconds

Shaping

Trial length = 4 seconds

Tone onset time = 1 second

Tone duration = 1 second

Conditioning water probability = 20%

Conditioning water onset time = 1.5 seconds

Conditioning water duration = .5 seconds

Inter-trial interval range = 5-9 seconds

Training block duration (should always be 1 hour) = 1 hour

Inter-block interval (should always be 1 hour) = 1 hour

No behavioral response time before each trial = 5 seconds

Early window = 0-1 seconds

Response window = 1-3 seconds

Hit water duration = 2 seconds

Tone Detection

Trial length = 4 seconds

Tone onset time = 1 second

Tone duration = 1 second

Inter-trial interval range = 5-9 seconds

Training block duration (should always be 1 hour) = 1 hour

Inter-block interval (should always be 1 hour) = 1 hour

No behavioral response time before each trial = 5 seconds

Early window = 0-1 seconds

Response window = 1-3 seconds

Hit water duration = 2 seconds

Time out duration = 20 seconds

Tone Discrimination

Trial length = 4 seconds

Tone onset time = 1 second

Tone duration = 1 second

Inter-trial interval range = 5-9 seconds

Training block duration (should always be 1 hour) = 1 hour

Inter-block interval (should always be 1 hour) = 1 hour

No behavioral response time before each trial = 5 seconds

Early window = 0-1 seconds

Response window = 1-3 seconds

Hit water duration = 2 seconds

Time out duration = 20 seconds
